# Distinct fibroblast functions associated with fibrotic and immune-mediated inflammatory diseases and their implications for therapeutic development

**DOI:** 10.12688/f1000research.143472.1

**Published:** 2024-01-10

**Authors:** Alexander M. S. Barron, Thomas Fabre, Saurav De

**Affiliations:** 1 Inflammation & Immunology Research Unit, Pfizer, Inc., Cambridge, Massachusetts, 02139, USA

**Keywords:** Fibroblast, fibrosis, inflammation, autoimmunity, therapy, cytokine

## Abstract

Fibroblasts are ubiquitous cells that can adopt many functional states. As tissue-resident sentinels, they respond to acute damage signals and shape the earliest events in fibrotic and immune-mediated inflammatory diseases. Upon sensing an insult, fibroblasts produce chemokines and growth factors to organize and support the response. Depending on the size and composition of the resulting infiltrate, these activated fibroblasts may also begin to contract or relax thus changing local stiffness within the tissue. These early events likely contribute to the divergent clinical manifestations of fibrotic and immune-mediated inflammatory diseases. Further, distinct changes to the cellular composition and signaling dialogue in these diseases drive progressive fibroblasts specialization. In fibrotic diseases, fibroblasts support the survival, activation and differentiation of myeloid cells, granulocytes and innate lymphocytes, and produce most of the pathogenic extracellular matrix proteins. Whereas, in immune-mediated inflammatory diseases, sequential accumulation of dendritic cells, T cells and B cells programs fibroblasts to support local, destructive adaptive immune responses. Fibroblast specialization has clear implications for the development of effective induction and maintenance therapies for patients with these clinically distinct diseases.

## Introduction

What pushed you over the edge, huh? I bet it started with something small: a little too much to drink, sitting in traffic getting a lung-full of diesel exhaust or a drafty window in your apartment. Maybe your boss starts making fun of you like that bully did when you were in middle school. Whatever the insult, it’s not a big deal if it only happens once. You shake it off. But it just … keeps … happening. Once you could take. But this? No. You broke, and you broke bad. And now we’re here in this grimy room that smells like cheap, week-old coffee and donuts made from dirty gym socks. Neither of us wants to be here, and we don’t have to be for long. I just want to know two, simple things: why are we here and how did you do it?

Well, this isn’t simple. Not all criminals are the same. Some are serial killers or arsonists, while others are burglars or blackmailers. It’s not just people that break bad; fibroblasts do it, too, and in just as many ways. They can be like Avon Barksdale, Marlo Stansfield or Bodie enabling neutrophils, macrophages and other innate immune cells to be the Bubbles of fibrotic diseases (FD). Or they can be like Viktor Bout and Yevgeny Prigozhin, organizing and arming forces of T, B and dendritic cells (DCs) in immune-mediated inflammatory diseases (IMID). Arson isn’t too different from how fibroblasts destroy articular cartilage in rheumatoid arthritis (RA). Poe’s Montresor immured Fortunato which, in a way, is what fibroblasts do in FD. As many ways in which people commit crimes, so too can fibroblasts be pathogenic in FD and IMID. In this review we will largely focus on IMID and sterile FD not associated with tumors. Although fibroblasts interact with many types of cells, this review will largely cover their relationships with leukocytes and the extracellular matrix (ECM). ^1^^–^^13^ By the end of this, you’ll know some of the reasons why fibroblasts break bad, what drives them do it and a few of the questions we still need to answer.

## Recruitment and positioning of specialized leukocytes

Leukocyte infiltrates are common across diseases that clinically manifest as either FD (e.g. idiopathic pulmonary fibrosis (IPF), non-alcoholic steatohepatitis (NASH) and systemic sclerosis (SSc)) or IMID (e.g. rheumatoid arthritis (RA), Sjogren’s syndrome (SS) and discoid lupus erythematosus (DLE)). However, the magnitude, composition, density and organization of leukocyte infiltrates differ. Generally, the number of infiltrating leukocytes per mm ^3^ appears to be lower in FD than IMID biopsies. Infiltrates in FD are generally dominated by innate immune cells like neutrophils and macrophages with smaller proportions of innate and tissue resident lymphocytes. IMID generally feature lymphocyte-rich infiltrates and aggregates. However, relatively few studies have directly compared the frequencies of lymphocyte aggregates between FD and IMID, and there is considerable histological heterogeneity. ^14^^–^^22^ This comparison is further complicated in FD by changes in the composition and size of infiltrates as tissues become more fibrotic. ^23^

**Figure 1.**
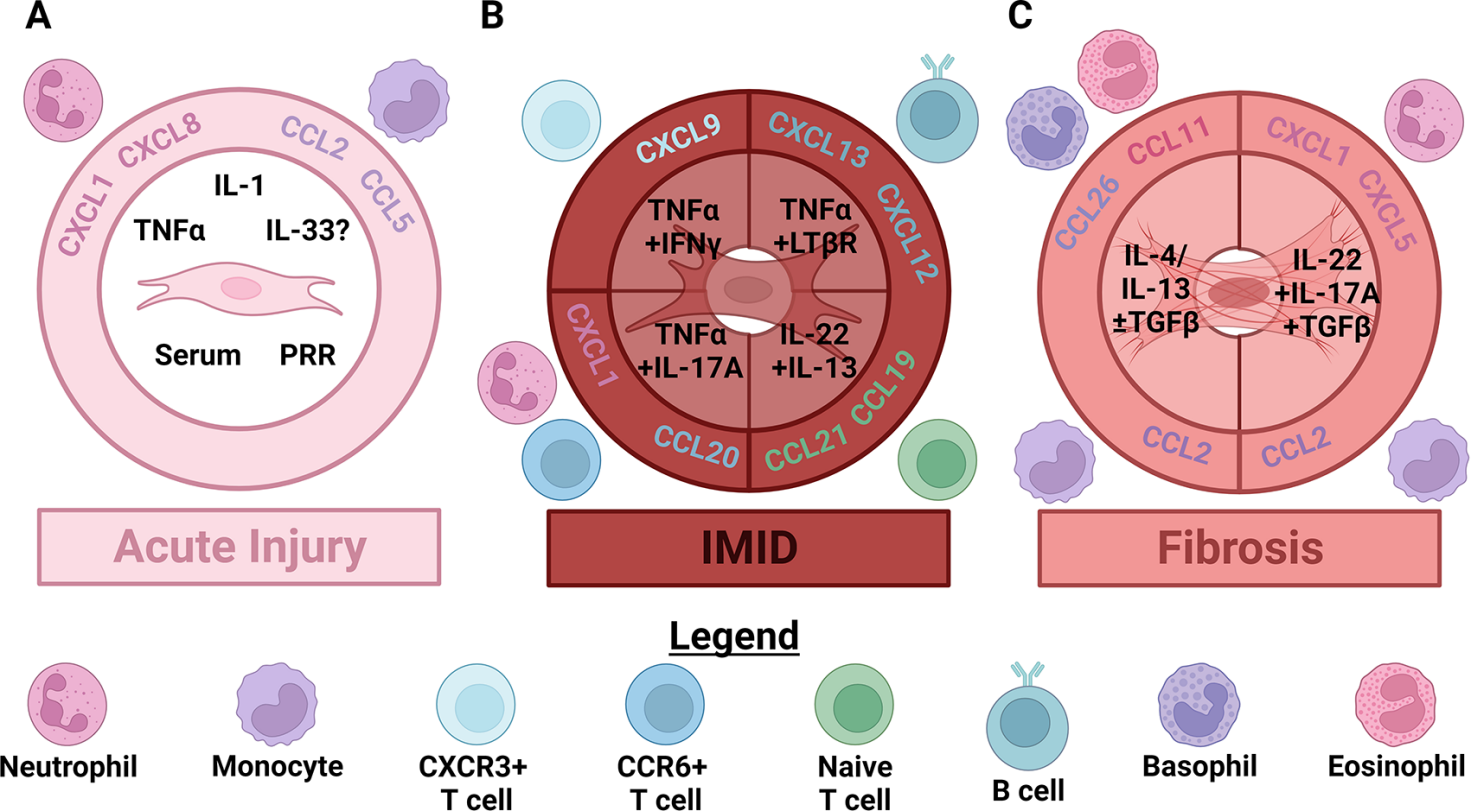
As acute injury progresses to chronic disease fibroblasts orchestrate changes in the size and composition of leukocyte infiltrates by producing distinct chemokines. (A) In acute injury, damage signals like serum, pattern recognition receptor (PRR) activation, interleukin (IL)-1 and tumor necrosis factor α (TNFα) activate fibroblasts. Whether fibroblasts are directly activated by IL-33 is still controversial. Fibroblasts activated by these signals produce chemokines that attract neutrophils like CXCL1 and CXCL8, as well as chemokines that attract classical monocytes like CCL2 and CCL5. (B) In immune-mediated inflammatory diseases (IMID) activated innate lymphocytes (including innate lymphoid, γδ T, NK, iNKT and MAIT cells) begin producing polarizing cytokines. This initiates local production of specialized chemokines which induce extravasation of the first polarized, memory αβ T cells that increase polarized cytokine production. In type 1 skewed responses this first wave of lymphocytes produces TNFα and IFNγ, which synergistically stimulate fibroblast secretion of CXCL9, CXCL10 and CXCL11. These cytokines attract more CXCR3+ type 1 or type 3 skewed T cells. IFNγ also appears to inhibit TNFα-induced production of neutrophil chemokines by fibroblasts. Fibroblasts instead stimulated by type 3 cytokines including TNFα and IL-17A continue secreting neutrophil chemoattractants like CXCL1 and begin producing additional cytokines like CCL20. These cytokines attract additional CCR6+ type 3 skewed T cells. Accumulation of enough activated dendritic cells and T cells leads to significant TNFα/TNF receptor 2 and lymphotoxin α1β2/lymphotoxin β receptor (LTβR) signaling. Activation of these pathways in fibroblasts can induce an additional stage of specialization that recruits naïve αβ T cells via secretion of CCL19 and CCL21, and B cells through CXCL13 and/or CXCL12. (C) Relatively few αβ T cells accumulate in sterile fibrotic diseases (compared to IMID) leading to distinct signaling and fibroblast differentiation. In fibrotic diseases largely characterized by type 2 skewing, IL-4 and/or IL-13, especially in concert with active TGFβ, induce secretion of CCL11, CCL26 and CCL2 by fibroblasts. CCL11 attracts eosinophils, CCL26 attracts basophils and CCL2 continues to attract classical monocytes. Conversely, in fibrotic diseases that exhibit more type 3 skewing IL-22 and IL-17A, in combination with TGFβ stimulate secretion of neutrophil chemokines, like CXCL1 and CXCL5, and CCL2 by fibroblasts. Note that most human IMID and fibrotic diseases are characterized by mixed, rather than purely polarized, inflammation. Figure created with BioRender.

Regardless of the insult or injury that initiates FD or IMID, the earliest events are likely shared between these diseases. These likely include the release of alarmins, damage associated molecular patterns (DAMPs) and a local increase in vascular permeability that results in a serum response (( Figure 1A) reviewed in Refs. 24– 26). Endothelial and myeloid cells express a wide repertoire of pattern-recognition receptors (PRRs) that recognize DAMPs, but fibroblast PRR expression appears to be more tissue-specific (reviewed in Ref. 27). ^3^^,^^28^^–^^32^ Fibroblasts are more widely responsive to alarmins and serum. ^33^^–^^48^ Although tissue-specific fibroblast responses to PRR signaling likely contribute to the sites and outcomes of FD and IMID, we focus on the more universal alarmin response here. Among the alarmins that fibroblasts respond to are IL-1α, IL-1β, and IL-33.

Measuring alarmins in patients is complex due to the lack of early diagnostics, heterogeneity in disease severity and course, medications, genetic background and other factors. Despite these challenges, increased expression of alarmins is associated with both FD and IMID although data from patient samples is relatively scarce. Increased IL-1β, IL-33 and TSLP protein was detected in the bronchoalveolar lavage fluid (BALF) of patients with IPF and SSc, although there was considerable variability between cohorts. ^49^^–^^53^ Higher levels of IL-1α were also observed in the serum from some SSc patients. ^54^ SSc patients treated with bermekimab, an IL-1α antagonist antibody, in a 12-week phase 2A trial had better lung function and fewer swollen joints supporting a pathogenic role for this cytokine in some patients. ^55^ Synovial fluid from RA patients had higher levels of IL-1β than osteoarthritis patients, although the detected levels were lower than TNF and IL-6. ^56^^–^^58^ This difference in local expression may partly explain the modest ACR score improvements in RA clinical trials of Anakinra compared to TNFα antagonists and tocilizumab. ^59^^,^^60^ Higher serum levels of IL-33 were observed in a subset of RA patients, which was associated with pulmonary complications and more severe disease. ^61^^,^^62^ Elevated synovial fluid IL-33 also distinguished RA patients from those with osteoarthritis. ^62^^,^^63^ These data suggest that alarmins can activate fibroblasts in either FD or IMID and may be pathogenic, although the biomedical community would benefit from higher-powered studies in genetically diverse patient cohorts.

IL-1α is expressed by various cell types in the cytoplasm and nucleus and can be released during cellular damage without any modification. In contrast, immature IL-1β must be processed by a specialized complex known as the inflammasome. The inflammasome is a supra molecular organizing complex comprised of adapter proteins and active caspases. Formation of the complex results in caspase mediated cleavage of IL-1β and the gasdermins that form the membrane pores required for the release of mature IL-1β (reviewed in Ref. 26). Both IL-1α and IL-1β bind to IL-1R1 on fibroblasts to elicit secretion of a variety of chemokines. Stimulation of fibroblasts with recombinant IL-1α *in vitro* induced CXCL8 RNA within 30 minutes and secretion of bioactive CXCL8 and CCL2 within 24 hours ( Figure 1A). Conditioned media from damaged lung epithelial cells induced transcription of these same chemokines, which required IL-1α, not IL-1β. In this case, IL-1α-driven transcription of these chemokines synergized with TLR3 signaling. This required at least JAK2, TAK1 and IKK2. ^36^^,^^44^^,^^45^ IL-1β induced production of CXCL8 and CCL2 as well as CXCL1 and CCL5/RANTES ( Figure 1A). ^46^^,^^48^ Together, these data show that fibroblasts from multiple sites can respond to the alarmins IL-1α and IL-1β by secreting neutrophil and monocyte chemokines.

Do these *in vitro* findings translate *in vivo*? Alum injection demonstrated that IL-1α ^-/-^ mice showed a partial decrease in neutrophil infiltration into the site of injection, while IL-1α ^-/-^ IL-1β ^-/-^ mice showed complete ablation of neutrophil infiltration indicating some redundancy between these cytokines. ^64^ Murine myocardial infarction results in large monocyte and neutrophil infiltrates into the damaged heart, which required IL-1 signaling in non-myeloid cells. ^65^ Conditional deletion of IL-1R1 using the *Col1a2*-CreER(T) line in this model reduced cardiac leukocyte infiltration, although the authors did not specifically quantify neutrophil or monocyte infiltrates. ^47^ Acute, diphtheria toxin-mediated depletion of Kupffer cells similarly demonstrated that IL-1α, IL-1β and TNFα activate hepatic stellate cells (fibroblastic cells found in the liver) and liver sinusoidal endothelial cells. Both cell types rapidly produced *Ccl2* to recruit monocytes and replenish the Kupffer cell niche. ^66^ Together these results suggest that IL-1α and IL-1β can stimulate fibroblast-mediated recruitment of neutrophils and monocytes *in vivo.* These data suggest that chemokine production by fibroblasts is important for monocyte and granulocyte recruitment in response to IL-1. However, a direct demonstration would require conditional, fibroblast-specific IL-1R1 knockout and quantification of monocyte and granulocyte infiltration.

Less is known about fibroblast responses to IL-33 than IL-1α and IL-1β. Human and murine fibroblasts can express IL-33 *in vivo* and *in vitro*. ^62^^,^^67^ *^–^* ^76^ As a nuclear and secreted factor that undergoes proteolytic processing, IL-33 biology is complex and requires further experimental clarification (reviewed in Ref. 77). The current picture is that homeostatic murine adventitial fibroblasts in several organs and differentiated lymphoid organ fibroblasts express IL-33 *in vivo*. ^67^ *^–^* ^69^^,^^71^^,^^72^ Either this is different for human fibroblasts or IL-33 expression decreases with traditional fibroblast culture methods: human fibroblasts required stimulation to induce IL-33 expression *in vitro.* Combined stimulation by TNFα and IFNγ triggered IL-33 expression by dermal fibroblasts, which was further enhanced by TLR3 activation. ^76^ Either TNFα or IL-1β alone stimulated IL-33 production by synovial fibroblasts from RA patients, which also synergized with TLR3 activation. ^74^^,^^75^ However, secreted IL-33 was rarely detected in these experiments. ^73^^–^^76^ Does this mean that fibroblasts express nuclear IL-33 but cannot secrete it?

It is possible that, under the conditions used in these studies, the cultured fibroblasts did not secrete IL-33. However, because conditional deletion of IL-33 from fibroblasts and ST2 from leukocytes *in vivo* yielded similar phenotypes, it seems likely that fibroblasts can secrete IL-33. ^78^^,^^79^ There are two alternative explanations that are also plausible. First, oxidization of IL-33 after secretion prevents binding to its receptor IL1RL1/ST2 and subsequent signaling. Distinct antibodies are required to detect oxidized IL-33 versus reduced IL-33. ^80^ Thus, the lack of detectable secreted IL-33 in these studies may have been due to this conformational change. This, however, seems less likely than the second alternative explanation. Alternative splicing of IL1RL1 yields both transmembrane and soluble proteins. The soluble form antagonizes IL-33 signaling. ^81^ Proteolytically processed IL-33 is a small protein (~175-161 amino acids). ^82^ Although not conclusively demonstrated for most IL-33 antibodies, it seems likely that the binding of IL-33 to soluble IL1RL1 would cross-block subsequent detection by anti-IL-33 detection agents. Production of soluble IL1RL1 by fibroblasts would thus impair detection of secreted IL-33 from these cells. Further empirical dissection of IL-33 secretion by fibroblasts would provide useful clarification.

Regardless of whether fibroblasts secrete IL-33 they can express the protein. Since the most robust evidence is for intracellular expression of IL-33, does this form exert effects on fibroblasts? When expressed in the nucleus, full-length IL-33 appears to repress transcriptional activation by NF-κB. This inhibition translated to reduced secretion of CXCL8 and CCL2 by RA synovial fibroblasts stimulated with TNFα alone or the combination of TNFα, IL-1β and a TLR3 agonist. ^74^^,^^75^ Thus, induction of intracellular IL-33 expression by IL-1 and TNFα may act as a negative feedback regulator of acute inflammatory responses by fibroblasts. Many cells aside from fibroblasts can secrete processed, highly active IL-33. Is there evidence that fibroblasts are impacted by this secreted form?

Data from RA synovial fibroblasts suggests that exogenous IL-33 may have the opposite effect from that of intracellular IL-33. Stimulation with recombinant IL-33 increased secretion of CXCL8 and transcription of several other genes. ^73^ Blocking IL1RL1 inhibited induction of some of these by IL-33 suggesting that fibroblasts may be activated by IL-33. ^74^ However, IL-33 also increased transcription of IL-1β in RA synovial fibroblasts. ^73^ As IL-1β can stimulate many of these same transcripts it is currently unclear which effects were the direct result of IL-33 signaling and which were indirectly stimulated by IL-33-driven IL-1β production ( Figure 1A). This is somewhat controversial as another study was unable to detect direct effects of exogenous IL-33 on the RA synovial fibroblasts they used. ^75^ All in all, it seems likely that fibroblasts can respond to IL-33 under some circumstances, however the effects and relative importance of this require further work.

Correlating with the elevated alarmin expression in patients with FD and IMID, levels of the alarmin-stimulated chemokines CCL2, CCL5, CXCL2 and CXCL8 were also higher patients with FD and IMID than healthy controls. ^83^^–^^88^ In addition to producing these chemokines in response to IL-1α and IL-1β, fibroblasts can produce these when stimulated with TNFα ( Figure 1A). This is true for human cells and animal models of both FD and IMID and occurs both *in vitro* and *in vivo*. ^17^^,^^22^^,^^85^^,^^89^^,^^90^ And in patients with inflammatory bowel disease (IBD) a fibroblast IL-1 response signature is correlated with local neutrophil infiltrates and resistance to both TNF antagonists and steroids. ^37^ This supports the redundance of IL-1 and TNFα in a subset of patients. These patients would likely benefit from combination therapy targeting these redundant signaling systems. If alarmins and DAMPs are produced in both FD and IMID, why are the infiltrate compositions generally so different between theses distinct clinical disease manifestations? The answers to these questions are currently unclear, although they likely involve the results of combinatorial signaling events, which will be explored next.

During the priming phase of antigen-specific responses, including IMID, immature DCs and tissue-resident macrophages are also activated by alarmins and PRR. Activated perivascular macrophages secrete chemokines like CXCL2 that initially attract maturing DCs towards blood vessels. Without an influx of activated antigen-specific T cells (or the presence of antigen-specific tissue resident T cells), these maturing DCs leave the damaged tissue through the draining lymphatics and prime antigen-specific naïve T cells in lymph nodes. ^91^^–^^93^ Similarly, in sterile FD where tolerance appears to be maintained any DCs that are activated during the initial insult likely migrate to the draining lymph nodes but fail to induce a T cell response. ^94^^,^^95^ How these discrepant responses occur is an interesting question, but beyond the scope of this review. Simultaneously, neutrophils and monocytes are drawn into the tissue by the chemokines produced in response to alarmins, PRR signaling and/or the serum response as described previously.

At this early stage, the monocytes likely differentiate into a mixture of macrophages and DCs. In IMID, the activated T cells proliferate, differentiate, exit the lymph node and enter the damaged tissue through activated post-capillary venules. ^96^^,^^97^ These T cells are restimulated by perivascular DCs presenting cognate antigens, and these reactivated T cells retain the DCs in the affected perivascular or peri-damaged space. ^91^^,^^98^^–^^103^ Retention of these DCs and the initial accumulation of activated antigen-specific T cells likely leads to nucleation of lymphocyte aggregates and infiltrates where lymphocytes predominate. Conversely, the lack of antigen-specific T cell influx and DC retention in FD leads to an entirely different set of signals. This, we propose, is where fibroblast activation states and functions in FD and IMID diverge.

Within hours of damage or insult fibroblasts in non-lymphoid tissues turn off their homeostatic programs and begin expressing Podoplanin (PDPN). ^104^^,^^105^ PDPN expression by human fibroblasts from multiple sites is induced by acute inflammatory stimuli like IL-1β and TNFα, consistent with this activation occurring prior to reparative or fibrotic signals like TGFβ. ^104^^,^^106^^,^^107^ Unliganded PDPN initiates fibroblast contraction by binding to ezrin, activation of Rho kinases (ROCK) and phosphorylation of ezrin/radixin/moesin family proteins and myosin light chain. ^108^^–^^110^ Fibroblast contraction can activate latent TGFβ through integrin αV heterodimers. Active TGFβ is both a suppressor of generally anti-fibrotic type 1 immune skewing and one of the most potent pro-fibrotic signals. ^111^^–^^116^ Therapeutic inhibition of integrin mediated latent TGFβ activation in fibrotic diseases is being pursued by multiple companies including Pliant Therapeutics. ^117^ Although TGFβ suppresses signals associated with type 1 immune polarization it appears to spare production of myeloid and neutrophil chemokines by fibroblasts, at least as part of the serum response and in combination with TLR4 or IL-17A. ^118^ Contraction also increases the local stiffness of the ECM which can initiate durotaxis and tension responses through integrins. ^119^

Durotactic attraction between myofibroblasts and macrophages is believed to be more effective than chemotaxis and haptotaxis. ^119^^,^^120^ Macrophages are especially important collaborators in generating the fibrotic niche: genetic or chemical depletion of macrophages abrogates fibrosis progression. ^121^^–^^128^ Once in proximity fibroblasts and macrophages establish strong adhesion through homotypic Cadherin-11 (CDH-11) contacts. This creates a pro-fibrotic niche where latent TGFβ produced by macrophages can be activated by myofibroblast contraction. ^129^ Thus, therapies aimed at limiting mechano-sensing in fibroblasts and macrophages are currently being explored. These include inhibition of PDPN, ROCK1/2 and focal-adhesion kinase (FAK) as well as strategies to dissociate fibroblasts from macrophages (e.g. CDH-11 antagonists). Immune cells other than macrophages can also sense mechanical forces, which similarly impact their activation and function. ^130^^,^^131^ Therefore, it is possible that neutrophils, eosinophils and others are recruited to the pro-fibrotic niche through the same mechanisms. Similarly, there are mechanisms other than PDPN that activate fibroblast contractility which likely play important roles in the fibrotic activation of fibroblasts. ^132^^–^^135^ Thus, the combination of contraction-induced durotaxis and selective sparing of myeloid and granulocyte chemokine expression already appears to differentiate fibroblast functions in FD from those in IMID.

Unlike the macrophages and neutrophils that predominate in fibrosis-associated leukocyte infiltrates, DCs express the PDPN ligand CLEC-2, which decouples PDPN from ezrin and inhibits fibroblast contraction. ^108^^,^^109^^,^^136^ This is one way that early differences in the composition of leukocyte infiltrates may result in different fibroblast functions and clinical pathologies in IMID and FD. Whether this is true *in vivo* requires experimental validation, though, as CD177, which is expressed by neutrophils, binds to PDPN and may exert similar effects. ^110^ Relaxation of fibroblasts around injured areas through interaction with DCs is likely necessary to accommodate early infiltration of T cells like the swelling and expansion of lymph nodes during immune responses. ^9^^,^^10^^,^^108^^,^^109^^,^^137^^–^^139^ In addition, these DCs may provide TNFR2 or LTβR ligands to trigger further fibroblast specialization. ^140^^–^^145^ Some sites, like the lung, seem to require local retention of DCs for sufficient secondary fibroblast activation and lymphocyte accumulation regardless of whether the response is type 1 or type 3 skewed. ^146^^–^^152^ In others, like the salivary glands, IL-22 and IL-13 produced by γδ and iNKT cells and ILC2s may be sufficient to initiate lymphocyte accumulation and secondary fibroblast activation. Sustained lymphocyte recruitment in this case appears to require an influx of IL-22 producing αβ T cells. ^107^^,^^153^^,^^154^ Regardless of the site and activating signals, this second round of fibroblast specialization in IMID seems to involve fibroblast proliferation and the induction of T cell recruiting chemokines by these cells. ^105^^,^^107^^,^^146^^–^^159^

Patterns of lymphocyte infiltrates can vary from diffusely spread throughout the tissue to tightly aggregated. ^14^^,^^160^^–^^163^ Aggregates frequently form around microanatomic structures such as blood vessels and pulmonary airways. ^14^^,^^67^^,^^163^^,^^164^ Among diseases with T cell aggregates, their size and organization can vary between small (~5-20), medium (~20-50) and large (>50). ^14^^,^^162^ Small T cell aggregates can likely be recruited by activated perivascular DCs without the help of specialized fibroblasts, but this may differ between mice and humans. ^14^^,^^91^^,^^98^ Growth of T cell aggregates beyond ~10 cells appears to require the expansion of specialized fibroblast networks. ^14^^,^^91^^,^^156^^–^^158^^,^^164^ As polarized, activated memory T cells enter an inflamed tissue, or as ILCs and other tissue-resident lymphocytes are activated, the combination of innate and adaptive cytokines likely tunes the fibroblast chemokine program to attract the appropriate lymphocytes for the response.

In the case of type 1 polarization, the synergistic combination of TNFα or IL-1 and IFNγ suppresses neutrophil chemokines *in vitro* while increasing production of CXCR3 ligands via cooperation between STAT1 and Nuclear factor-κB (NF-κB, Figure 1B). ^165^^–^^169^ Fibroblasts expressing CXCL9 are important for CXCR3+ T _H_1 differentiation in lymph nodes, and similarly activated fibroblasts expressing multiple CXCR3 ligands are increased in the salivary glands of SS patients, synovia of RA patients and in tumors. ^70^^,^^170^^–^^174^ Likely these activated fibroblasts contribute to extravasation of type 1 or type 3 lymphocytes expressing CXCR3 ( Figure 1B). ^98^^,^^175^^–^^178^ Conversely, combined stimulation of fibroblasts with the type 3 stimuli TNFα and IL-17A decreased CCL3 and CCL5 secretion while increasing expression of CCL20 and neutrophil chemoattractants. ^90^^,^^179^^–^^181^ Whether a similar fibroblast state occurs in patients awaits experimental verification. These fibroblasts are likely adapted to draw in type 3 polarized CCR6+ lymphocytes and neutrophils ( Figure 1B). ^178^^,^^182^ Specialized adventitial fibroblasts can indirectly instigate type 2 lymphocyte recruitment by creating specialized niches for ILC2s and eosinophils. ^67^ These observations suggest an evolving dialogue between leukocytes and fibroblasts that can recruit specialized lymphocytes in IMID with distinct immune polarization. The relative importance of fibroblast production of these chemokines requires experimental investigation *in vivo.*

The recruitment of polarized lymphocytes by fibroblasts appears to occur early in the growth of aggregates. Small lymphocyte aggregates are dominated by CD45RO+ T cells, which have likely been polarized during their activation in secondary lymphoid organs (SLO). Further fibroblast specialization seems to be required to recruit B cells and naïve T cells as the frequency of B cells and naïve T cells increases with aggregate size. ^14^^,^^156^^,^^157^^,^^164^ Extravasation and localization of naïve T cells into SLO requires secretion of the CCR7 ligands CCL19 and CCL21 by fibroblasts. ^70^^,^^183^^–^^187^ Similar fibroblasts expressing CCL19 and CCL21 emerge in several IMID and tumors, and also appear to correlate with T cell infiltration and lymphoid aggregate size. ^21^^,^^156^^–^^158^^,^^171^^,^^188^^–^^192^ In patients with interstitial pneumonia with autoimmune features, BALF CCL19 concentrations and lymphocyte numbers were positively correlated supporting CCL19-induced pulmonary lymphocyte infiltration. Longitudinal tracking of these patients demonstrated that high baseline BALF CCL19 and lymphocytes were associated with a lack of pulmonary fibrosis up to one year later. This observation supports divergent fibroblast activation occurring in IMID versus FD, and that the signals that induce these states may cross-inhibit each other. ^193^ What induces fibroblast production of CCL19 and CCL21 in IMID is also unclear, but the clues we have are discussed below.

In lymphoid organs, there is evidence for TNFR1/2 and LTβR ligands driving expression of these chemokines ( Figure 1B). Pharmacological or genetic antagonism of LTβR signaling reduces or prevents CCL21 and CCL19 expression in models of IMID. ^107^^,^^140^^,^^141^^,^^145^^,^^184^^,^^192^^,^^194^^–^^197^ However, expression of CCL21 in the lungs appears to be independent of both TNFR1/2 and LTβR signaling. ^198^ We are unaware of *in vitro* confirmation of CCL19 and CCL21 induction in fibroblasts by these signals. Larger reductions in *CCL19* RNA were seen in RA patients with EULAR responses to disease-modifying anti-rheumatic therapies (DMARDs) than those who failed to respond, which suggests a potential association with pathogenesis. ^199^ But whether fibroblasts drive significant disease pathology by producing these chemokines remains an open question.

B cell infiltrates into IMID tissues are generally less common than T cell infiltrates. ^14^^,^^156^^,^^188^ However, in some IMID like SS, RA and DLE, large B cell infiltrates occur in substantial proportions of patients (approximately 42% in RA, 89% in SS and 92% in DLE) although some of these cohorts were relatively small. ^14^^,^^156^^,^^157^ Aggregates containing both T and B cells can either be non-segregated, with T and B cells evenly distributed throughout, or segregated, with discrete T cell zones and B cell follicles similar to SLO. ^14^^,^^21^^,^^156^^,^^157^^,^^164^^,^^192^^,^^200^^,^^201^ In this review we define “tertiary lymphoid structures” (TLSs) as the subset of lymphocyte aggregates with these discrete T cell zones and B cell follicles. Infiltration of B cells in IMID, especially smaller infiltrates that lack T-B cell separation, occur in the absence of (or preceding) mature follicular dendritic cells (FDC, B cell supporting fibroblasts in SLO). ^14^^,^^156^^,^^157^ Regardless of FDC maturation status, CXCL13 generally appears necessary for inflammation-associated increases of B cells in peripheral tissues ( Figure 1B). ^156^^,^^157^^,^^202^^–^^206^ However, in specific cases like the lung B cell infiltrates can occur in the absence of CXCL13 and appear to require CXCL12 ( Figure 1B). ^150^ This could suggest that CXCL12 is sufficient for B cell extravasation and localization, or that CXCL13 negative pulmonary TLSs are exclusively populated by resident memory B cells. ^207^^,^^208^

In IMID where CXCL13 is induced, expression by myeloid and peripheral helper T cells (T _PH_) is likely sufficient to drive the initial accumulation of a few memory B cells. ^156^^,^^157^^,^^202^^,^^203^^,^^209^ As inflammation progresses, however, expression of CXCL13 by fibroblasts appears important for generating local germinal centers. ^14^^,^^156^^,^^157^^,^^162^^,^^203^^,^^210^ During SLO development, CXCL13 expression can be induced by or independent of TNFR1/2 and LTβR signaling depending on the site and timing ( Figure 1B). ^145^^,^^195^^,^^211^^–^^213^ Similarly mixed observations have been made in murine models of IMID and lymphoid aggregate formation. Antagonizing LTβR signaling was sufficient to reduce or inhibit CXCL13 production in the non-obese diabetic (NOD) model of SS and a model of autoimmune pancreatitis. ^192^^,^^194^^,^^197^ IL-22 production is necessary for induction of CXC13 by salivary gland and lung fibroblasts, but the salivary gland induction depends on LTβR while the lung does not. ^154^^,^^214^ Together, these data suggest that a stepwise dialogue between leukocyte infiltrates and fibroblasts causes fibroblast differentiation that may separate IMID from FD.

Alarmins induced neutrophil and monocyte chemokine secretion by fibroblasts as well as contraction-induced durotaxis. This first fibroblast activation state is likely similar between IMID and FD ( Figure 1A). Next, in IMID a first wave of activated, polarized T cells infiltrates the tissue resulting in local retention of DCs and fibroblast relaxation. Collaboration between TNFR and LTβR ligands produced by DCs with adaptive cytokines produced by T cells, ILCs, NK, MAIT and iNKT cells results in a second step of fibroblast differentiation. Second-stage IMID fibroblasts likely begin to express CCL19, CCL21, CXCL12 and CXCL13, which recruits B cells and naïve T cells ( Figure 1B). Perhaps, as recently described, ligation of ICOSL on fibroblasts or CD40/CD40L-dependent T _PH_/B cell interactions are necessary for further lymphoid aggregate expansion and compartmentalization. ^196^^,^^215^ Conversely, in FD the monocyte- and neutrophil-rich infiltrates promote fibroblast contractility and stiffening of the matrix. This leads to the activation of latent TGFβ, which combines with alarmin signaling to continue monocyte and neutrophil extravasation while preventing T cell infiltration ( Figure 1C). From these observations, divergent paths of fibroblast activation between FD and IMID begin to emerge.

## Rationing of growth factors

From the previous section it appears that fibroblasts can contribute to differential leukocyte extravasation in FD and IMID. However, the number of cells that can occupy a tissue is regulated by the availability of growth and survival factors. ^216^^–^^219^ This observation raises our next two questions. First, having contributed to leukocyte infiltration, do fibroblasts provide survival signals to these cells in FD and IMID? The answer, as this section will demonstrate, appears to be yes. These survival signals can take the form of soluble factors, transmembrane molecules or modifications of the ECM. We will concentrate on secreted growth factors in this review. Second, do fibroblasts sense and regulate cell composition by changing the types and quantities of growth factors they provide? This is an active area of research, but initial data suggests that they do.

In line with the correlation between elevated alarmin levels and monocyte and granulocyte chemokines in FD and IMID, growth factors to support these innate leukocytes are also elevated in some diseases. GM-CSF but not G-CSF was higher in synovial fluid of RA versus OA patients, and there appeared to be an inverse relationship between synovial fluid GM- and G-CSF concentrations. ^220^^–^^222^ Levels of GM-CSF correlated with IL-1β and TNFα quantities in the synovial fluid of RA patients, and both IL-1β and TNFα induced secretion of GM-CSF by synovial fibroblasts. ^222^ GM-CSF protein levels were higher in urine from patients with active lupus nephritis compared to inactive systemic lupus erythematosus, other chronic kidney diseases and healthy controls. ^223^ G-CSF was detectable in tears from both IgG4-related disease and SS patients, however levels in SS patients did not appear to differ from healthy controls. ^224^^–^^226^ Data on growth factor levels in local fluid and biopsy samples in FD is scarcer than for IMID. What is available shows a trend towards increased GM- and G-CSF in some patients, which was positively correlated with neutrophils in these samples. ^18^^,^^227^^–^^230^ Although the quality and amount of data varies considerably between diseases, increased in growth factor quantities were generally associated with increased acute damage signals and monocyte and neutrophil chemokines in these patients. Might fibroblasts contribute to the production of these growth factors?

**Figure 2.**
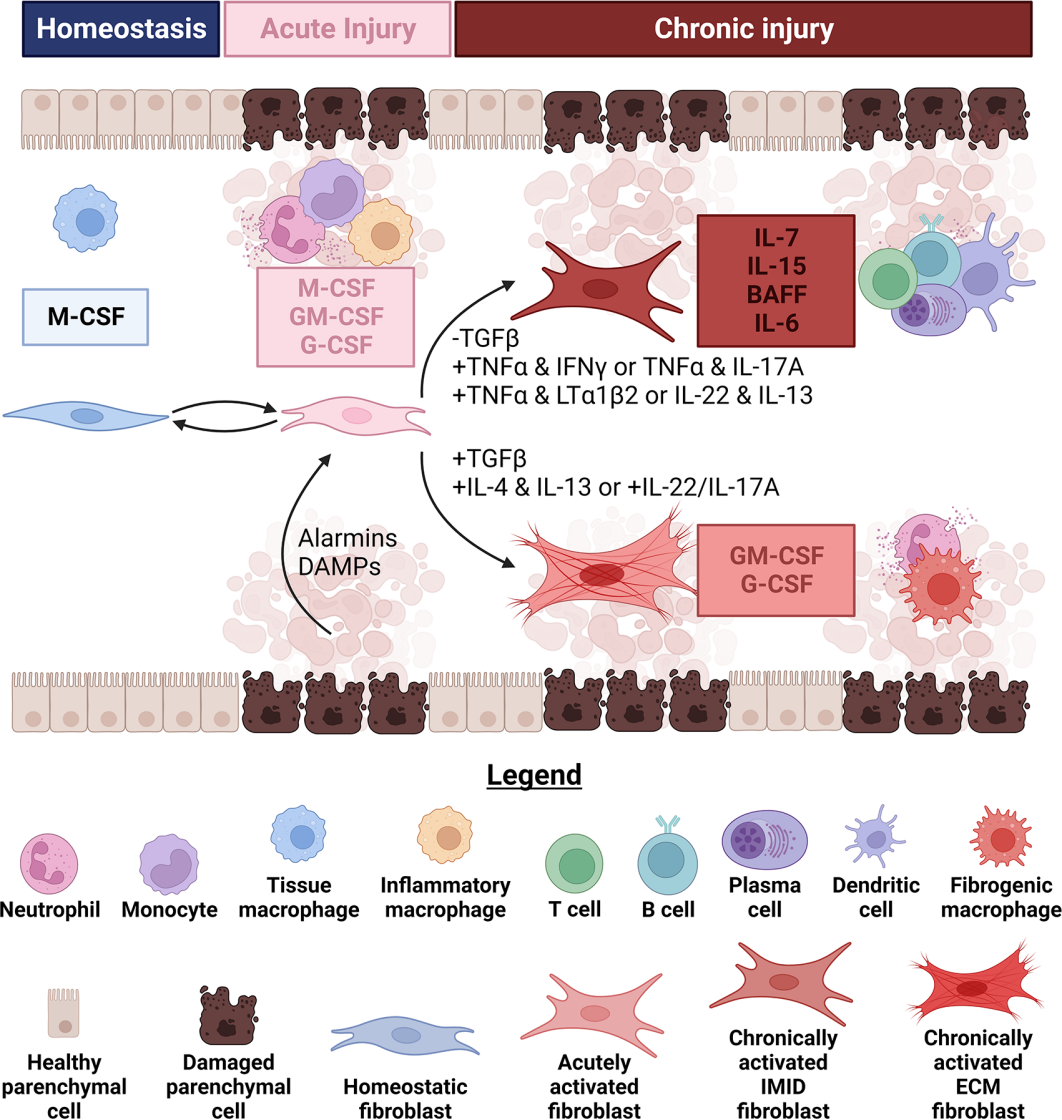
Fibroblasts influence changes in leukocyte infiltrate size and composition by altering the types and amounts of growth factors. During homeostasis, fibroblasts in several lymphoid and non-lymphoid tissues produce growth factors (generally Csf1/macrophage colony stimulating factor (M-CSF)) to sustain tissue-resident macrophage populations. Immediately upon injury, fibroblasts increase the amount of growth factors they secrete and begin producing Csf2/granulocyte-macrophage colony stimulating factor (GM-CSF) and Csf3/granulocyte colony stimulating factor (G-CSF). Increases in the amounts and breadth of growth factors produced by fibroblasts coincide with their elevated secretion of chemokines that attract granulocytes, monocytes and macrophages (described in subheading 1). Alarmins, damage-associated molecular patterns (DAMPs) and serum from leaking blood vessels can each stimulate these functional changes in fibroblasts. These acute damage signals can also synergize to further enhance the quantities of growth factors produced by fibroblasts. If the injury is successfully contained, fibroblasts can help replenish the depleted tissue-resident macrophage population by attracting monocytes and secreting M-CSF to induce their differentiation. Conversely, if damage signals persist chronic fibrotic (FD) or immune-mediated inflammatory diseases (IMID) can result partly because of divergent fibroblast functional states. Retention of dendritic cells (DCs) and infiltration of type 1 or type 3 polarized αβ memory T cells lead to progressive differentiation of fibroblasts in response to combinations of cytokines like tumor necrosis factor α (TNFα) and interferon γ (IFNγ) or TNFα and IL-17A. DCs can also relax fibroblasts, which decreases contractile activation of latent transforming growth factor β (TGFβ). Mature DCs or activated lymphocytes can produce TNFα and lymphotoxin α1β2 to initiate a second round of fibroblast specialization, triggering secretion of growth factors like IL-7 and IL-15 for T cells and BAFF and IL-6 for B and plasma cells. Fibroblasts appear to reach similar functional states in response to IL-22 and IL-13 in at least one murine model of IMID, although how these cytokines can induce such distinct fibroblast states in FD and IMID is currently unclear. Specialized fibroblasts producing IL-7, IL-15, BAFF and IL-6 likely underpin the formation of large lymphocyte aggregates or tertiary lymphoid structures in IMID. On the other hand, activation of latent TGFβ through several mechanisms in FD, including by contractile fibroblasts, synergizes with IL-4 and IL-13 or IL-22 and IL-17A to maintain production of GM-CSF and G-CSF. Both support monocyte differentiation and granulocyte survival within the fibrotic niche. Figure created with BioRender.

Fibroblasts from various lymphoid and non-lymphoid tissues produce growth factors for myeloid cells and granulocytes, although other cell types including endothelial cells can also play significant roles. ^66^^,^^231^ Specialized fibroblasts in the murine lymph node expressed both Csf1/M-CSF and IL-34, the two Csf1r ligands, *in vivo* and induced expansion of classical monocytes and macrophages *in vitro*. ^70^^,^^232^^,^^233^ Fibroblasts in the murine splenic red pulp appeared to serve a similar function as they also expressed both *Csf1* and *Il34*. ^234^^,^^235^ Targeted deletion of *Csf1* in splenic red pulp, but not white pulp, fibroblasts dramatically reduced red pulp macrophages ( Figure 2). ^235^ These data support the importance of fibroblast-derived growth factors for maintenance of myeloid cells during homeostasis. What about after injury?

One-time depletion of homeostatic murine macrophages by diphtheria toxin induced a controlled, acute inflammatory response. *Csf1* expression was induced in hepatic stellate cells (fibroblastic cells of the liver) upon acute depletion of liver resident Kupffer cells. Repopulation of the Kupffer cell niche by monocytes required M-CSF-Csf1r signaling. In this model, however, *Csf1* was expressed by both stellate cells and liver sinusoidal endothelial cells in response to IL-1α, IL-1β and TNFα. The relative importance of *Csf1* expression by each cell type remains an open question ( Figure 2). ^66^ A similar observation was made after acute depletion of GATA6+ cavity macrophages. Here, both mesothelial cells and capsule fibroblasts provided M-CSF. ^236^ Again, the relative contributions of these two cell types to M-CSF production awaits experimental validation in this model. Regardless of their relative importance, these data indicate that fibroblasts play significant roles in the homeostatic maintenance of tissue macrophage and the restoration of tissue macrophage populations after acute injury. As we saw in the chemokine section above, fibroblasts responded to the alarmins produced in acute injury by attracting both monocytes and granulocytes. Like M-CSF, do fibroblasts supply Csf2/GM-CSF or Csf3/G-CSF to support these infiltrating innate immune cells?

*In vivo* many different cell types produce GM-CSF. Unlike the M-CSF-dependent tissue macrophages described in previous paragraphs, alveolar macrophages require GM-CSF in both humans and mice. ^237^^–^^244^ Multiple cell types expressed GM-CSF in the homeostatic murine lung including type 2 innate lymphoid cells (ILC2), γδ T cells and alveolar type 2 epithelial cells (AT2). However, only GM-CSF from AT2 was required for alveolar macrophage differentiation and maintenance. ^245^ The critical source of CM-CSF may differ between sites, and likely changes in disease. For example, in patients with various forms of inflammatory arthritis fibroblasts, endothelial cells and macrophages all appear to produce GM-CSF. ^246^ To some extent this is recapitulated in the murine SKG arthritis model where GM-CSF production by fibroblasts and ILC2 is necessary for disease, but not in the serum-transfer induced arthritis model where GM-CSF from NK cells is required ( Figure 2). ^247^^,^^248^ The critical sources of G-CSF *in vivo* during homeostasis and insult or disease are less clear, although production by endothelial cells is necessary for TLR4-driven emergency granulopoiesis. ^249^ These data indicate that fibroblasts may be important sources of GM- and G-CSF in IMID and FD, but that further work is necessary to determine this *in vivo.*

*In vitro* quiescent, growth inhibited human dermal fibroblasts produce almost no leukocyte growth factors. They respond to acute serum stimulation alone (in the absence of alarmins or other exogenous cytokines) by producing mostly bioactive M-CSF (little to no GM- and G-CSF). Stimulation with IL-1α induced secretion of M-, GM- and G-CSF with amounts of M->GM->G-CSF. Acute serum and IL-1α stimulations demonstrated additive synergy in the production of all three growth factors ( Figure 2). ^250^ Human lung fibroblasts also secreted M-CSF in response to acute serum stimulation. TNFα stimulation demonstrated additive synergy with serum in the induction of M-CSF, and triggered GM-CSF production. Co-treatment with the corticosteroid dexamethasone inhibited the induction of GM-CSF but not M-CSF by TNFα suggesting a potential mechanism of action for this steroid. ^251^ This growth factor program appears to be conserved as TNFα, IL-1β, IL-17A or IFNγ each increased the ability of synovial, bone marrow and skin fibroblasts to support neutrophil survival *in vitro, which largely* depended on GM-CSF. ^90^^,^^222^^,^^252^ Thus, fibroblasts activated by alarmins or serum in FD and IMID may contribute to the local survival of monocytes, macrophages and granulocytes although this may differ by tissue or disease ( Figure 2). Further work is necessary to determine how important the production of these growth factors by fibroblasts is and will benefit greatly from the use of genetically engineered mice allowing inducible, fibroblast-specific deletion of these growth factors.

Acute injury signals appear to be shared between FD and IMID. What leads to the expression of differentiated growth factor programs by fibroblasts translate into these divergent clinical manifestations? As acute injuries become chronic diseases, new signals arise with the changing cellular composition and continuing damage. Layering these new signals on top of chronic alarmin, serum and PRR signaling, and the cross-repression between these signals, is what seems to modify the growth factor programs of fibroblasts. This kind of cross-repression is common during organ development and immune cell differentiation. ^1^^,^^67^^,^^72^^,^^253^ Studies of how sequential or simultaneous signals regulate the provision of growth factors by fibroblasts in adulthood are a newer undertaking with many open questions. Investigating these more complex interactions is, however, necessary to understand human diseases which are often characterized by mixed inflammatory and morphogenic signals. ^115^^,^^254^

TGFβ is one of the dominant cytokines mediating fibroblast activation and fibrosis in multiple tissues (reviewed in Refs. 255, 256). It is also a potent suppressor of some types of immune responses (reviewed in Refs. 257, 258). Given the relatively small size of leukocyte infiltrates in most FD (compared to IMID), and the predominance of myeloid cells and granulocytes, an obvious question is: does combining TGFβ with pre-existing alarmin, PRR or serum signaling suppresses the production of M-CSF, GM-CSF or G-CSF by fibroblasts? To our knowledge no publications have directly explored this question, but two pieces of evidence suggest that TGFβ may not suppress production of these survival factors. First, TLR4 knockout mice are protected from hepatic fibrosis, which correlates with lower levels of TGFβ signaling in hepatic stellate cells, reduced production of macrophage and neutrophil chemoattractants by hepatic stellate cells and reduced macrophage infiltration into injured livers. ^118^ The reduction in both hepatic stellate cell TGFβ signaling and hepatic macrophage infiltration in TLR4 knockouts argues against TGFβ suppressing the production of myeloid survival factors by HSC. Second, serum or IL-1 alone stimulates the secretion of M-CSF, GM-CSF and G-CSF from human fibroblasts, and the combination of serum plus IL-1 demonstrates additive synergy. ^250^ The 10% FBS-supplemented media that induced secretion of these three CSFs by fibroblasts likely contained ≥1000 pg/mL of bovine TGFβ, which can be activated by freezing and thawing and has bioactivity on human cells. ^250^^,^^259^^,^^260^ Thus, it seems likely that fibroblasts continue to produce myeloid and granulocyte growth factors to support key constituents of the fibrotic niche when exposed to TGFβ, although this needs to be tested *in vitro* and *in vivo* ( Figure 2).

What about IMID where many patients have large increases of T and B cells that are frequently found in aggregates? IL-7 protein levels were higher in the synovial fluid of some RA patients compared to OA patients and it was locally transcribed in the synovium. ^261^^,^^262^ Levels of IL-15 protein in synovial fluid of RA patients were also higher than OA patients in some cohorts, and this was also transcribed in the synovium. ^262^^–^^265^ Fibroblasts isolated from the synovial tissue of RA patients spontaneously secreted more IL-7 and IL-15 than OA synovial fibroblasts. Production of both IL-7 and IL-15 from RA synovial fibroblasts increased in response to IL-1β or TNFα suggesting a role for NF-κB. ^262^^,^^266^ IL-7 was detectable in tears from both IgG4-related disease and SS patients, however levels in SS patients did not appear to differ from healthy controls. ^224^^–^^226^ IL-7 and IL-15 protein levels were also higher in urine from patients with active lupus nephritis compared to inactive systemic lupus erythematosus, other chronic kidney diseases and healthy controls. ^223^ Do fibroblasts provide these growth factors in IMID to support the observed increases in lymphocytes?

Small aggregates predominantly comprised of T cells are likely sustained by IL-15 presented by myeloid and endothelial cells, IL-2 from locally proliferating T cells and IL-7 produced by lymphatic endothelial cells. ^91^^,^^267^^–^^272^ Medium and large aggregates, though, appear to require the expansion and differentiation of specialized fibroblasts. ^14^^,^^137^^,^^273^ Although we currently lack a definitive mechanistic explanation for this observation, specialized fibroblasts likely control the size of T cell aggregates in several ways. Fibroblastic reticular cells (FRC), which are found in the T cell zones of secondary lymphoid organs (SLO), directly control T cell numbers by limiting or increasing survival signals such as IL-7 and IL-15. ^184^^,^^185^^,^^269^^,^^274^^–^^276^ The mechanisms by which fibroblasts are activated to produce IL-7 and IL-15 are still unclear. Genetic ablation of LTβR in *Ccl19*+ lymph node fibroblasts reduced *Il7* expression, and transgenic overexpression of LTβR ligands in the pancreas induced *Il7* suggesting that LTβR signaling may be sufficient. ^184^^,^^192^ However, pharmacologic or genetic LTβR antagonism increased *Il7* transcripts in bone marrow fibroblasts. ^277^

Fibroblast regulation of T cell numbers in primary (PLO) or SLO through production or restriction of growth and survival factors like IL-7 seems likely. ^184^^,^^185^^,^^274^^,^^278^^–^^280^ Expansion of specialized fibroblasts that produce similar T cell survival factors is likely required to increase the carrying capacity of inflamed tissues and support medium and large aggregates ( Figure 2). ^14^^,^^105^^,^^107^^,^^185^^,^^191^^,^^252^^,^^268^^,^^281^ However, despite our presentation of circumstantial evidence for this hypothesis it has yet to be directly tested. Rigorously testing this hypothesis is challenging as fibroblasts express similar transcriptional programs across tissues, and tissue-specific fibroblast modules are often shared with non-fibroblast cells in those same tissues. ^70^^,^^174^^,^^190^^,^^282^ Conditional deletion is clearly necessary to avoid confounding developmental defects, as seen in the constitutive, fully-body IL-7 knockout. ^283^ To untangle effects specific to the inflamed non-lymphoid tissue from potential effects of growth factor deletion in PLO or SLO fibroblasts, we propose orthotopic adoptive transfer of purified fibroblasts from mice genetically modified for conditional growth factor deletion or parabiosis between wild type and mice with fibroblast-specific conditional growth factor deletions. ^105^^,^^284^ These experiments could be complemented by spatial transcriptomics or combined in situ hybridization and protein staining in biopsies from human inflammatory diseases to show neighborhood-based enrichment of IL-7 expression specifically by fibroblasts scaffolding large T cell aggregates. Testing for cross-species conservation is critical given the differences in production and presentation of growth and survival factors (e.g. IL-15) and fibroblast behavior between species. ^14^^,^^270^^–^^272^^,^^285^^,^^286^

Specialized fibroblasts in primary and secondary lymphoid organs are major producers of the B cell growth factors IL-6, BAFF and APRIL, as well as the marginal zone B cell survival factor Delta-like 1. ^70^^,^^233^^,^^287^^–^^291^ In fact, BAFF is expressed by multiple fibroblast subsets in lymph nodes: one within B cell follicles, one in the interfollicular T cell zones and one in the medullary cords. ^233^^,^^287^^,^^292^^,^^293^ These microanatomically distinct fibroblasts are also functionally different with some supporting B cells and others supporting plasma cells. ^287^^,^^293^ Increased BAFF expression has been detected in the affected joints of RA patients and in the affected glands of SS patients. ^107^^,^^294^ For both RA and SS patients with lung complications, increased BAFF expression is also detected in patients with pulmonary B cell-containing lymphoid aggregates. ^21^ Local production of survival factors appears especially important to support autoreactive B cells in IMID. Autoantigen-specific B cells are particularly sensitive to activation-induced death due to chronic B cell receptor (BCR) stimulation, and BAFF can rescue these autoreactive B cells through non-canonical NF-κB-mediated Pim2. ^295^ Simply observing increased production of B cell growth factors in IMID does not necessarily mean these are being produced by fibroblasts, though. Myeloid cells and granulocytes are also capable of producing many of these proteins. ^296^^–^^300^ What evidence supports these factors coming from activated fibroblasts?

There are two lines of evidence. First, specialized fibroblasts producing BAFF transcripts have been identified by scRNAseq in dissociated joints from RA patients and salivary glands from SS patients ( Figure 2). ^107^^,^^189^ Second, synovial fibroblasts isolated from RA patients secrete BAFF in response to TLR3 or TNFα stimulation and constitutively secrete APRIL. ^143^^,^^301^ Matched dermal fibroblasts from the same RA patients also produced BAFF transcripts in response to TLR3 and TLR4 stimulation, but secreted ~10-fold less BAFF and APRIL than synovial fibroblasts even after stimulation. ^301^ Production of these growth factors by fibroblasts is effective as synovial fibroblasts support B cell survival *in vitro* in co-cultures. ^302^ Whether BAFF expression increases prior to B cell infiltration as a prerequisite for B cell infiltration, or pioneer B cells induce BAFF to create a feed-forward loop is an open question. Based on kinetic studies in a murine model of SS where elevation of BAFF transcripts appears slightly before B cell infiltration we propose that early alarmin signaling by tissue resident lymphocytes is necessary to induce TLR3/4 and/or TNFR2 expression by fibroblasts. In turn, activation of tissue resident lymphocytes, either by fibroblasts or other cells in close proximity, induces surface expression of TNFα or LTα homotrimers to trigger TNFR2-driven non-canonical NF-κB activation and BAFF transcription in fibroblasts ( Figure 2). ^107^^,^^153^^,^^154^^,^^196^^,^^301^^,^^303^ These data suggest a tissue- or inflammation-driven epigenetic and post-transcriptional regulation program that differentiates RA synovial and dermal fibroblasts, and may partly account for the accumulation of B cells in affected joints but not unaffected skin of these patients.

Together, these data suggest that fibroblasts may provide critical growth factor support for leukocytes infiltrating tissues affected by FD and IMID. In these tissues, fibroblasts appear to adopt distinct activation or differentiation states that evolve as acute insults become chronic diseases with distinct clinical manifestations. This increasing fibroblast specialization seems to result from progressive signaling dialogues that differ between fibrotic diseases and IMID. Provision of specific collections of growth factors by IMID or FD fibroblasts may be a result of these different combinations of signals and a mechanism to control the size and composition of leukocyte infiltrates. We propose that this is necessary for the different clinical manifestations and organs affected by various FD and IMID. Our theory is that cross-repression and synergistic induction of receptors and genetic modules in fibroblasts drive these distinct fibroblast functions, pathology and tissue tropism in IMID and FD.

## Masters of the extracellular matrix (ECM)

When thinking about the pathogenic functions of fibroblasts, the most obvious starting point is fibrosis. Fibrosis, or scarring, is the result of abnormal repair in response to chronic injury. The molecular and cellular mechanisms that cause fibrosis are complex, but removal of healthy, homeostatic ECM and its replacement by pathologic fibrillar collagen is a major part of these diseases. Fibroblasts are at the center of this process, and what usually comes first to mind is the myofibroblast. Conventionally, the definition of a myofibroblast includes two components: production of fibrillar collagens and other ECM proteins, and contractility which is usually shorthanded to α-SMA+ fibroblasts (reviewed in Ref. 304). ^134^ We agree with the long-form definition of “myofibroblast”. However, activated fibroblasts that lack detectable α-SMA protein express fibrillar pro-collagen proteins and are associated with areas of fibrosis in some diseases. ^305^ Thus, we prefer the more general term “ECM fibroblast” when referring to activated fibroblasts in FD with myofibroblasts being a subset of ECM fibroblasts.

Fibrosis can occur in chronic diseases with either type 2 or type 3 immune polarization (or in the absence of obvious inflammation or leukocyte infiltration), but this clear-cut skewing is more common in animal models than patient cohorts. More frequently, human fibrotic diseases demonstrate varying degrees of mixed inflammation. Due to cross-repression and rebound inflammation, stopping progressive scarring in patients with obvious inflammatory skewing will likely require simultaneous antagonism of both type 2 and type 3 signaling. ^115^^,^^116^^,^^254^^,^^306^ Even more complicated, the predominant inflammatory skewing of FD patient cohorts likely varies depending on the organ and stage of disease in the biopsy. For example, the lungs of active IPF patients generally have three distinct zones: relatively healthy, inflamed with little fibrosis and overtly acellular due to end-stage fibrosis (reviewed in Ref. 307). Depending on the zone sampled researchers will get wildly divergent results. Although this example is true for IPF, similar caution is warranted when interpreting findings from other fibrotic diseases. The extent and predominance of type 2, type 3 or a lack of overt inflammation among patients with different fibrotic diseases is an open area of research requiring larger patient cohorts of diverse genetic backgrounds.

IL-4, IL-5 and IL-13 are the canonical cytokines associated with type 2 inflammation. ^308^ Increased levels of these cytokines have been detected in patients with a variety of sterile fibrotic diseases including NASH and IPF. Depending on the cohort and the way in which the measurements were taken, higher levels of these cytokines are associated with more severe and/or longer disease duration. ^115^^,^^254^^,^^309^^–^^311^ Fibroblasts isolated from the lungs of IPF patients express more of the IL-4 receptor α (IL-4Rα) and IL-13 receptor α1 (IL-13Rα1) than fibroblasts from healthy lungs. These two proteins form the heterodimeric type II receptor for IL-4 and IL-13, and this elevated expression was associated with increased *COL1* transcription in response to these cytokines. ^312^^,^^313^ IL-4 and IL-13 can also stimulate COL1 secretion by some human hepatic stellate cell lines. ^314^ However, the mechanisms by which these cytokines, especially IL-13, induce fibrosis may differ between organs or diseases.

**Figure 3.**
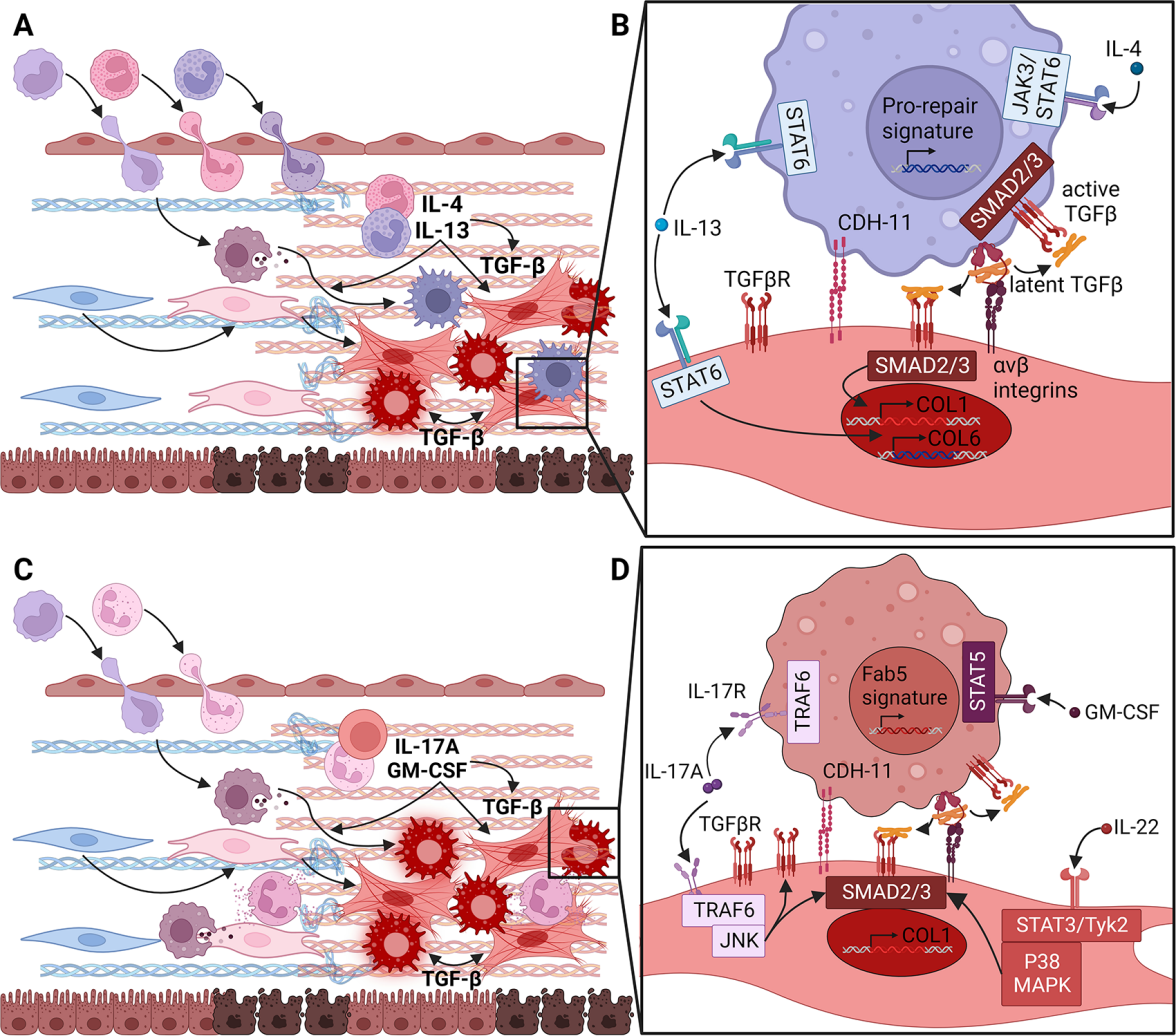
Type 2 or type 3 cytokines can differentiate extracellular matrix (ECM) fibroblasts, which form distinct fibrotic niches. (A) In predominantly type 2 skewed fibrotic diseases (FD), acutely activated fibroblasts and other cells attract monocytes, eosinophils and basophils. Degranulation of eosinophils and basophils can release IL-4 and IL-13 to activate the transcription factor STAT6. Eosinophils granules can also release TGFβ to promote differentiation of ECM fibroblasts into myofibroblasts through the transcription factors SMAD2 and SMAD3. IL-4 and IL-13 also influence monocyte differentiation into macrophages, which can then add to local TGFβ production and myofibroblast differentiation. (B) By signaling through the type II receptor, IL-4 and IL-13 can directly activate production of Collagen type VI (COL6) by fibroblasts. Thus, they may directly drive fibrosis without relying on TGFβ although in this situation it is unclear how production of fibrillar collagens is initiated. IL-4 and IL-13 can also stimulate TGFβ production by macrophages. Thanks to homotypic Cadherin-11 (CDH-11) synapses formed with myofibroblasts, the TGFβ produced by these macrophages can be converted from its latent to active form through fibroblast integrin αV heterodimers and contractile force. Thus, IL-4 and IL-13 may trigger Collagen type I (COL1) production by ECM fibroblasts through this indirect TGFβ pathway. (C) In FD dominated by type 3 inflammatory skewing, acutely activated fibroblasts and other cells primarily induce monocyte and neutrophil extravasation. Under these circumstances, γδ T cells, neutrophils and mast cells produce IL-17A and, with fibroblasts, GM-CSF. These induce the differentiation of infiltrating monocytes into Fab5 macrophages. IL-17A can also potentiate low levels of TGFβ signaling generated through contractile activation by ECM fibroblasts expressing unbound podoplanin (PDPN). (D) GM-CSF produced by fibroblasts and other cells initiates monocyte differentiation towards the Fab5 macrophage state through the transcription factor STAT5. As described in (B), Fab5 macrophages can also form CDH-11 synapses with ECM fibroblasts and provide latent TGFβ for activation by fibroblast integrin αV and contraction. Active TGFβ can augment or stabilize the Fab5 program in macrophages and promote ECM fibroblasts to become myofibroblasts. IL-17A potentially produced by several cell types in the fibrotic niche can increase expression of TGFβRII and enhance SMAD2 and SMAD3 activation by signaling through TRAF6 and JNK in fibroblasts. In differentiating monocytes, IL-17A can also promote differentiation into Fab5 macrophages. IL-22, another type 3 cytokine, can also enhance SMAD2 and SMAD3 activation in concert with TGFβ. Figure created with BioRender.

Liver fibrosis induced by local transgenic overexpression of IL-13 and signaling through the type II receptor on fibroblasts (including hepatic stellate cells) may be independent of TGFβ. ^116^^,^^315^^,^^316^ Pulmonary fibrosis induced by similar transgenic overexpression of IL-13 in murine lungs, although induced using a different genetic technique, required TGFβ for fibrosis ( Figure 3A). ^317^^,^^318^ Why this distinction was observed is currently unclear. Different results were obtained from the strongly type 2 polarized *Schistosoma mansoni* models of lung and liver fibrosis, though, with both appearing to be independent of TGFβ. ^116^^,^^316^ Perhaps ECM fibroblast activation and fibrosis in these models depends on the magnitude of the IL-13 response or the balance between mixed inflammatory polarization?

In a murine model of severe asthma characterized by strong, mixed type 2 and type 3 inflammation neither reducing TGFβ activation nor IL-13 blockade alone or in combination decreased fibrosis. ^116^ This last observation suggests two possibilities. First, that in the presence of multiple strong, non-redundant ECM fibroblast activating signals the degree of combined inhibition achieved insufficient target coverage. Second, that this model drives ECM fibroblast activation by an additional, orthogonal mechanism. Regardless of which is true, these data clearly demonstrate that type 2 inflammation, and specifically IL-13, can induce ECM fibroblast activation and fibrosis both directly and indirectly through TGFβ. One other part of this story is curious: IL-13 appears to directly stimulate *Col6a1* transcription via STAT6 activation in fibroblasts, but the mechanism by which IL-13 induces COL1 production is unclear ( Figure 3B). ^315^ Collagen type VI is not a traditional fibrillar collagen like types I, II and III but instead forms beaded microfilaments that are assembled into reticular structures (reviewed in Ref. 319). Perhaps dramatic increases in local Collagen type VI stiffen the local matrix and cascade through outside-in integrin signaling and activation of latent growth factors like TGFβ?

As noted above, type 3 inflammation is also associated with fibrosis. Type 3 inflammation is characterized by the recruitment and activation of T-helper (Th) 17 cells, γδ T cells, type 3 innate lymphoid cells (ILC3) and neutrophils ( Figure 3C). Infiltrates where these cells predominate plus local activation of mast cells leads to the secretion of IL-17A, IL-22 and GM-CSF ( Figure 3C). ^254^^,^^320^^–^^322^ Type 3 immune responses have been associated with fibrosis and cancer progression in many organs. IL-17A cytokine or receptor knockout animals are protected from fibrosis in variety of pre-clinical models. ^72^^,^^309^^,^^323^ Interestingly, opposing pro- vs anti-fibrotic functions have been reported for IL-22 depending on timing, type of injuries and opposite effects reported on epithelium vs mesenchymal cells. ^323^^–^^326^ Increased expression of IL-17A is associated with IPF, NASH and other fibrotic diseases while, to our knowledge, increased IL-22 has only been observed in NASH patients. ^254^^,^^309^ Elevated GM-CSF expression is also associated with NASH, IPF and mustard gas pulmonary fibrosis. ^229^^,^^230^ As we have described in previous sections, type 3 cytokines are associated with recruitment and survival of macrophages and granulocytes in FD, but also with the recruitment of DCs and lymphocytes in IMID. Do type 3 cytokines drive similarly opposing ECM depositing and eroding functions in fibroblasts?

IL-17A is the prototypical type 3 cytokine, and its receptor is expressed on a variety of cell types including epithelium, endothelium, mesothelium, and myeloid cells. ^327^ It signals through a heterodimeric receptor composed of the IL-17RA and IL-17RC subunits triggering a cascade through the Act1/TRAF6 to activate NF-KB, ERK1/2, C/EBPB, JNK and MAPK pathways (reviewed in Ref. 328). In the absence of serum, IL-17A only triggers secretion of pro-inflammatory chemokines in primary human hepatic stellate cells. In contrast, IL-17A sensitizes myofibroblasts to the action of TGFβ by stabilizing its receptor on the surface of cells in a JNK-dependent manner ( Figure 3C). ^329^ In conclusion, IL-17A is pleiotropic cytokine which impacts myofibroblast directly and/or indirectly within the fibrotic niche.

IL-22 is a complex pleiotropic cytokine from the IL-10 family, with opposite roles in health and disease. IL-22 signals through a heterodimeric receptor composed of the common IL-10 family chain IL-10RB and IL-22RA1 triggering activation of STAT3 and MAPK. ^330^^,^^331^ Expression of the IL-22 receptor complex is restricted to epithelial cells and fibroblasts. IL-22 promotes the survival and proliferation of epithelial cells via STAT3 promoting tissue repair and therefore limiting fibrosis. ^330^ On the other hand, IL-22 signaling also induces the secretion of chemokines involved in the recruitment of neutrophils, monocytes, and macrophages. ^332^^,^^333^ Studies in the liver also demonstrated that IL-22 contributes to fibrosis progression by enhancing TGFβ signaling via STAT3/MAPK activation ( Figure 3D). ^254^ While IL-22 deficient mice have impaired tissue repair mice lacking the decoy receptor IL-22BP also have impaired tissue repair due to aberrant inflammatory responses. These discrepancies require further investigation but likely depend on the levels of IL-22 and IL-22BP, the timing of IL-22 production, the cells receiving IL-22 signals and the other signals being simultaneously received and integrated.

Pathogenic production and organization of the ECM in fibrotic diseases is clearly one function of fibroblasts. Do they serve similar, but opposite, roles in inflammatory diseases characterized by excessive destruction of the ECM like RA? The answer appears to be yes, which is especially important when considering treatment for rheumatoid arthritis patients with the fibroid pathotype and osteoarthritis (OA) patients. Due to the low frequency of lymphocytes, myeloid cells and granulocytes in the joints of these patients next generation RA drugs should simultaneously block multiple pathways to cover patients with all three pathotypes. ^161^ Given the similarities between the fibroid RA pathotype and OA, these drugs may work for both groups of patients.

In rheumatoid arthritis mouse models, PDPN+FAPα+THY1- synovial fibroblasts specialize in joint destruction and extracellular matrix remodeling as demonstrated through engraftment. They secrete high levels of matrix metalloproteinases (MMPs) 3, 9 and 13, which are especially effective at degrading the most abundant fibrillar collagen in cartilage, Collagen type II, and the major constituent of basement membranes, Collagen type IV. ^171^^,^^284^^,^^334^ These PDPN+FAPα+THY1- synovial fibroblasts described by Croft *et al*. are likely the same as the *PRG4*+ synovial fibroblasts described by Micheroli *et al.* Both lack or have undetectably low expression of *THY1*, express *MMP3* and appear to be associated with the destruction of synovial cartilage independent of leukocyte infiltrates. ^284^^,^^335^

What activates these destructive functions in fibroblasts? IL-1, IL-17A, TNFα and Oncostatin M all induce combinations of MMPs in synovial fibroblasts, which result in degradation of glycosaminoglycans or cartilage *in vitro*. ^144^^,^^336^^,^^337^ Connecting MMP gene or protein expression with functional consequences is particularly important given the tight regulation of their activity. Pro-MMPs undergo proteolysis before becoming catalytically active, and after activation MMPs can be inhibited by the tissue inhibitor of metalloproteinases (TIMP) family of proteins (reviewed in Ref. 338). The balance between protease production, activation and suppression is also relevant for ECM fibroblasts. Clearance or modification of homeostatic ECM often accompanies the deposition of fibrotic ECM, and this requires the activation of specific classes of proteases and the inhibition of others. Although it is beyond the scope of this review, the generation of matricryptins or matrikines (proteolytic fragments of ECM molecules that induce signaling distinct from their larger, intact parent molecules) likely play roles in both fibrotic diseases and IMID (reviewed in Refs. 319, 339, 340). Despite the complexities of studying the pathogenic destruction of ECM by fibroblasts, these data indicate that fibroblasts are sufficient for clinically relevant joint destruction. Further research is necessary to determine whether fibroblasts make significant contributions to ECM destruction in other diseases.

It may be surprising that some of the type 3 cytokines associated with pathologic deposition and organization of ECM by fibroblasts can also induce the opposite outcome. For example, why is IL-17A associated with fibrosis in murine models and human NASH and IPF patients and with joint destruction in murine models and human RA patients? This is still an open question. However, these distinct pathologies and disease outcomes may be due to different signals combining with IL-17A in fibrotic versus eroding diseases. In FD, the combination of IL-17A and TGFβ signaling in fibroblasts can potentiate more robust ECM gene expression than either cytokine alone. ^329^ Add outside-in sensing of the stiff fibrotic matrix and hypoxia on top of the synergy between IL-17A and TGFβ, and this combination is likely part of what drives fibroblast to deposit pathogenic ECM in FD. There is less TGFβ and more TNFα signaling in RA to combine with IL-17A, which results in higher MMP13-driven proteoglycan destruction. ^179^^,^^336^ Similarly, how does IL-13 promote ECM fibroblast differentiation and fibrosis either alone or in combination with TGFβ in some models, while also driving fibroblast activation in TLS formation in a model of SS?

These data clearly demonstrate that distinct fibroblast activation or differentiation states and functions occur in FD and IMID. ECM fibroblasts are at the heart of FD. They are the major producers and organizers of collagens and many other ECM molecules, and tune the stiffness of tissues. By modifying the ECM in this way, fibroblasts also activate latent growth factors and convert haptotactic to chemotactic gradients. Are all ECM fibroblasts the same, or are there different functional states that arise from predominant type 2 versus type 3 activation (or in the absence of detectable inflammation)? And do all fibrotic fibroblasts deposit the same ECM constituents or exert the same contractile forces, or is collaboration between multiple ECM fibroblast states necessary for fibrosis? Answering these questions will be necessary for the rational development of therapeutics that stop or even reverse FD. For example, if the fibroblasts activated by type 3 cytokines build fibrotic ECM with a different composition from those activated by type 2 cytokines the molecular pathways requiring therapeutic manipulation will differ. This would also necessitate the development of companion diagnostics to effectively select patient populations for clinical trials and deliver the most effective personalized medicine to FD patients.

Conversely, different fibroblast subsets are responsible for ECM destruction or lymphocyte recruitment and support in RA. Thus, multiple fibroblast functional states can coexist and collaborate to drive complex disease pathology. Is ECM degradation by erosive fibroblasts necessary for the expansion of fibroblasts that scaffold lymphocyte aggregates and TLS? As thickening and stiffening of the lymph node capsule appears to be a compensatory reaction that constrains swelling and limits lymphocyte proliferation, some degradation of the local ECM is likely necessary for the growth of lymphocyte aggregates. ^108^^,^^109^^,^^139^ That erosive fibroblasts are sufficient for destruction of the collagen type II-rich cartilage in synovial tissues also begs the question of how important different cell types are to to ECM destruction. Similarly, if erosive fibroblasts in RA can significantly destroy the synovial matrix do they also make major contributions to removal of the healthy, homeostatic matrix in fibrotic diseases? Addressing these questions is critical to developing better therapies for patients with both erosive IMID and FD. Understanding the signals that create erosive versus ECM fibroblasts and whether these are stable differentiation states or reprogrammable activation states will help us develop the appropriate therapeutic regimens to treat these opposing disease pathologies.

### Subheading 4: Controlling leukocyte differentiation and activation in non-lymphoid tissues

Up to this point we have demonstrated that fibroblasts appear to adopt progressive, functionally distinct activation states in IMID versus FD. In IMID these various activated fibroblasts produce chemo-/haptotactic and growth factors specialized to support local adaptive immune responses. Conversely, in FD ECM and myofibroblasts generally attract smaller infiltrates of predominantly macrophages and granulocytes through specialized chemo-, hapto- and durotactic gradients and growth factors for these cells. Fibroblasts can also exert significant, opposing effects in these diseases by depositing or, at least in RA, degrading large amounts of ECM. The final question we will tackle is: do specialized fibroblasts contribute to the activation or differentiation of infiltrating leukocytes in these diseases?

**Figure 4.**
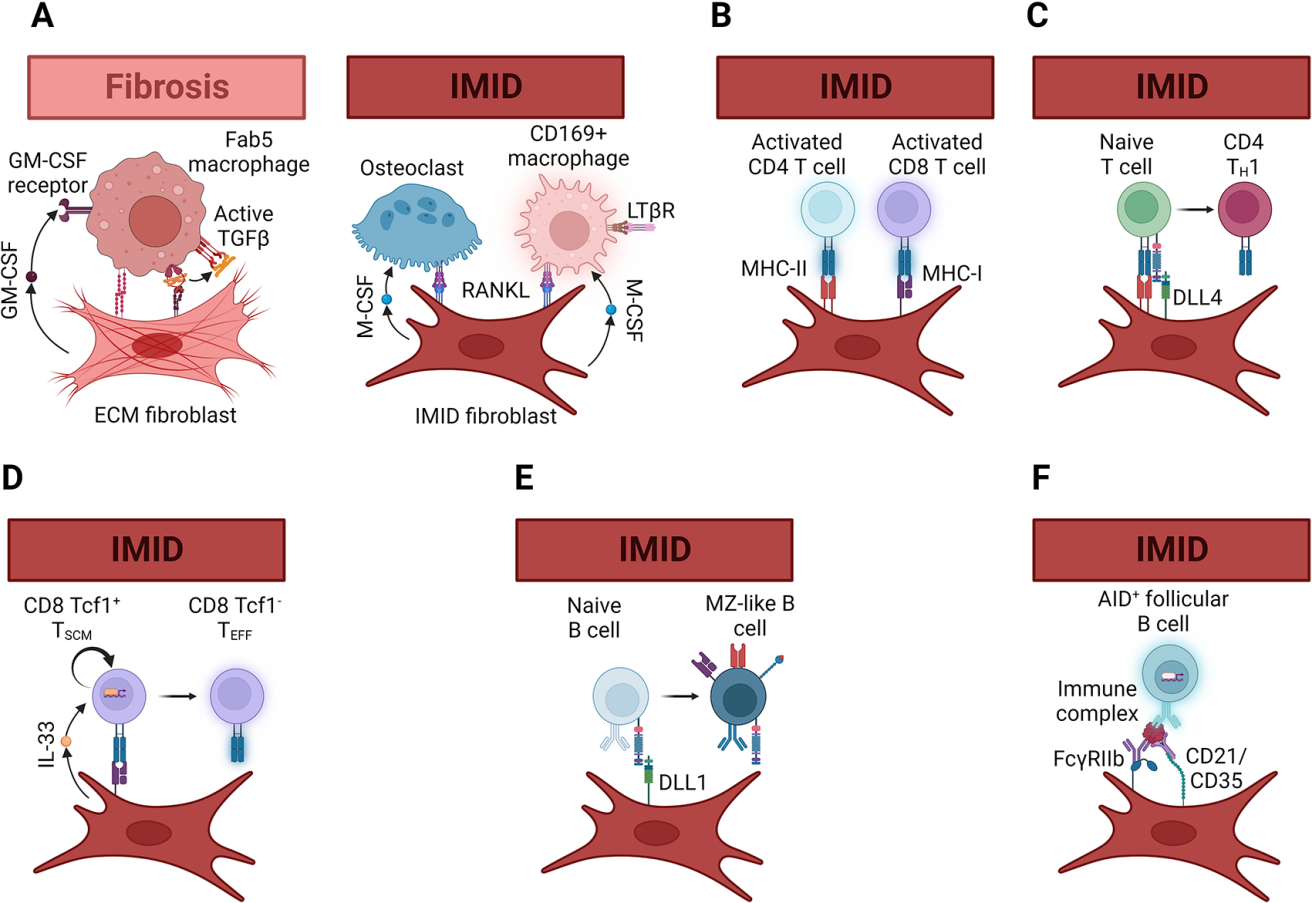
Fibroblast mechanisms to activate leukocytes in fibrotic and immune-mediated inflammatory diseases. (A) In fibrotic diseases, fibroblasts and macrophages can form Cadherin-11 mediated synapses. By secreting granulocyte-macrophage colony stimulating factor (GM-CSF) and activating latent transforming growth factor β (TGFβ) through integrin αV contractile forces, extracellular matrix (ECM) fibroblasts can promote differentiation of monocytes into fibrotic Fab5 macrophages. On the other hand, in immune-mediated inflammatory diseases (IMID) production of receptor activator of nuclear factor κB ligand (RANKL) and macrophage colony stimulating factor (M-CSF) can lead to osteoclast or CD169+ macrophage differentiation. Although the molecular mechanisms that dictate whether a macrophage becomes and osteoclast or CD169+ macrophage require further investigation, the dependence of CD169+ macrophage differentiation on LTβR signaling may be one differentiating factor. (B) Fibroblasts can express both major histocompatibility class-I (MHC-I) and -II (MHC-II), which can reactivate memory CD8 and CD4 T cells as they infiltrate tissues affected by IMID. Although not discussed in this article, MHC expression by fibroblasts can suppress T cell proliferation or promote tolerance in addition to restimulating T cells ^429^. (C) Fibroblasts in secondary lymphoid organs produce the Notch1/2 ligands Delta-like 1 (DLL1) and 4 (DLL4). In murine models of graft versus host disease, expression of DLL4 and, to a lesser extent, DLL1 by lymphoid organ fibroblasts was necessary for T _H_1 differentiation of grafted naïve T cells. This DLL1/4-Notch1/2-induced T cell differentiation was required for disease induction although it is unclear whether non-lymphoid organ fibroblasts can perform the same function. (D) IL-33 production by *Ccl19*+ is necessary for both maintenance of the Tcf1+ CD8 stem memory T cell pool and for effective anti-viral and anti-tumor CD8 T cell differentiation. The mechanism by which IL-33 produced by fibroblasts promotes type 2 immunity in parasite infections and type 1 anti-viral and anti-tumor immunity is unclear, but likely occurs through crosstalk with other signals like type 1 interferons. (E) DLL1-Notch2 signaling induces the differentiation and maintenance of splenic marginal-zone B cells. A similar system may work in tissues affected by IMID, but this awaits experimental testing. (F) Follicular dendritic cells in secondary lymphoid organs trap antibody- and complement-containing immune complexes using the Fcγ receptor IIb (FcγRIIb) and complement receptors 1 (CD35) and 2 (CD21). Presentation of the antigens in these immune complexes to B cells is important for germinal center reactions. Affinity maturation and class-switch recombination mediated by activation-induced cytidine deaminase (AID) in follicles both require this follicular dendritic cell function. Fibroblasts expressing FcγRIIb, CD35 and CD21 appear in the affected tissues of some IMID patients, and stimulation of fibroblasts isolated from these tissues can induce expression of these three receptors. Thus, it is likely that follicular dendritic-like cells can maintain germinal center reactions in IMID tissues. Figure created with BioRender.

Monocytes and macrophages are important parts of both IMID and FD, although they are a much larger proportion of infiltrates in FD than IMID. Recently, several reports identified scar-associated pro-fibrotic macrophages in human and mice in a variety of tissues. ^17^^,^^341^^–^^350^ A common signature (Fab5) using the combination of CD9, CD63, FAPB5, GPNMB and SPP1 was recently suggested to accurately distinguish these fibrotic macrophages from populations with other functions. Fab5 macrophages originate from monocytes and, in some settings, require IL-17A, GM-CSF (likely from neutrophils, although fibroblasts and other cell types may play a role) and TGFβ for their differentiation *in vitro* and *in vivo* ( Figure 3D). ^18^^,^^245^^,^^247^^,^^248^ Another route to a Fab5-like macrophage may be through M-CSF and IL-6, both of which can be produced by fibroblasts although the authors of this study identified an autocrine loop of these factors. ^351^ Fab5 macrophages elicit several key pro-fibrotic functions that were elucidated *in vitro.* Fab5 macrophages contribute to the removal of normal ECM through MMP activity and enhance deposition of fibrillar collagen by ECM fibroblasts ( Figure 3D). Although the molecular mechanisms by which Fab5 macrophages activate ECM fibroblasts are actively being studied, they likely include proximal presentation of latent TGFβ for activation by fibroblasts, as well as Amphiregulin and possible TWEAK and PDGFR ligands. ^17^^,^^129^^,^^352^ The relative importance of ECM fibroblast-produced M-CSF, GM-CSF, IL-6 and activation of latent TGFβ to Fab5 differentiation requires experimental testing, although it seems likely they contribute ( Figure 4A).

Fibroblasts likely induce differentiation of functionally distinct macrophage subsets in IMID. In SLO, differentiation of CD169+ sinusoidal and metallophillic (or marginal zone) macrophages requires Receptor activator of nuclear factor-κB ligand (RANKL/TNFSF11) expression by fibroblasts ( Figure 4A). These macrophages capture free virus and initiate anti-viral CD8 T cell responses. ^353^^–^^355^ The roles of CD169/SIGLEC1+ macrophages in IMID are unclear. One report on RA patients proposed a protective role for CD169+ macrophages, while others noted a positive correlation of circulating CD169+ myeloid cells with worse and extra-glandular disease in SS and dermatomyositis. ^356^^–^^358^ Given the recent reports on potentially pathogenic CD8 T cells, and reductions in synovial CD8 T cells being associated with clinical responses to rituximab in RA patients, we favor the hypothesis that CD169+ macrophages are pathogenic in IMID. ^161^^,^^171^^,^^359^^–^^363^

Several fibroblastic lineage cells in the bone and bone marrow induce osteoclast differentiation, thus indirectly promoting bone and cartilage resorption, by producing RANKL. ^364^^,^^365^ Do fibroblasts play similar roles in IMID-associated osteoporosis? PDPN+FAPα+THY1- synovial fibroblasts in RA patients and murine models produce RANKL, which promotes macrophage differentiation into osteoclasts and is associated with bone erosion. ^171^^,^^284^^,^^334^^,^^366^ Although RA appears to be the only IMID directly associated with bone erosion or osteolysis, many IMID are treated with glucocorticoids and long-term use of these steroids is a risk factor for osteoporosis (reviewed in Ref. 367). ^368^ Glucocorticoids can directly induce RANKL expression by fibroblastic cells from several tissues in mice and humans as the glucocorticoid receptor directly binds to the RANKL promoter. ^369^^,^^370^ Osteolysis also occurs in adverse responses to joint replacement and is frequently accompanied by histologic macrophage activation. ^371^^,^^372^ Both RANKL and its receptor, RANK, are expressed in tissues containing wear particles and osteoclasts spontaneous differentiated in patient samples with high RANKL to Osteoprotegrin (OPG, the soluble decoy receptor for RANKL) transcript ratios. ^373^ Titanium particles were sufficient to indirectly induce RANKL expression by synovial fibroblasts, as was combined stimulation with TNFα and IL-17A which worked through trans IL-6 signaling ( Figure 4A). ^374^^,^^375^ These data indicate a surprisingly wide range of osteolytic pathologies that appear to be driven by fibroblast-induced osteoclastogenesis.

Do fibroblasts participate in the local re-activation of T cells after they extravasate into inflamed tissues? Fibroblasts (both human and murine) can express Class I (MHC-I) and Class II (MHC-II) Major Histocompatibility Complex molecules. ^70^^,^^171^^,^^189^^,^^376^^–^^384^ Murine fibroblasts can activate both CD4 and CD8 T cells in an MHC-dependent fashion to elicit cytokine production and cytotoxic activity ( Figure 4B). ^377^^,^^378^^,^^380^^,^^383^^–^^385^ Human fibroblasts may be able to do the same, but definitive proof awaits experimental validation. ^379^^,^^382^ This could be demonstrated *in vitro* by obtaining donor-matched fibroblasts from skin biopsies and circulating T cells from the blood, pulsing the fibroblasts with a cocktail of common viral T cell epitope peptides and testing for proliferation and cytokine production. Given the suppression of effector T cell activation by primary human SLO fibroblasts and lymphocytic choriomeningitis virus clone 13 (LCMV-C13) infected murine fibroblasts, blocking co-inhibitory molecules either alone or in combination with CRISPR-mediated knockout of other suppressive factors may be necessary to detect much cytotoxic activity. ^385^^–^^390^ The relative importance of MHC expression by fibroblasts, macrophages and DCs in both SLO and IMID also await direct experimental validation. Is antigen presentation the only mechanism by which fibroblasts can restimulate infiltrating T cells?

No, although the relative importance of antigen-specific and -independent reactivation of T cells infiltrating inflamed, non-lymphoid tissues requires further investigation. Fibroblasts may reactivate infiltrating T cells through a variety of antigen-independent mechanisms including cytokine production, expression of canonical or non-canonical co-stimulatory molecules and adhesion proteins. SLO fibroblasts are important sources of the Notch ligands Delta-like 1 (Dll1) and 4 (Dll4) in both homeostasis and graft-versus host disease (GVHD, Figure 4C). ^281^^,^^290^^,^^391^^,^^392^ Homeostatic human dermal fibroblasts lack Notch ligands, but this may change (and not just in the skin) during inflammation as non-lymphoid fibroblasts adopt SLO-fibroblast-like qualities and awaits experimental testing. ^14^^,^^105^^,^^393^ If true, inflamed fibroblasts expressing Dll1 or Dll4 may reactivate infiltrating T cells to promote inflammatory or autoimmune tissue destruction ( Figure 4C). ^290^^,^^391^ Expression of Dll1 or Dll4 by inflamed fibroblasts may also promote local tertiary lymphoid tissue formation by driving differentiation of infiltrating naïve T cells into T follicular helpers and naïve B cells into germinal centers. ^290^^,^^394^ An alternative explanation is that Dll1 or Dll4 expression is truly restricted to SLO fibroblasts and is only important for imparting the ability to home to inflamed tissues, not the restimulation of tissue destructive effector functions. ^392^ It seems plausible, at least, that activated fibroblasts in IMID can contribute to inflammatory tissue destruction by reactivating infiltrating T cells and driving T cell differentiation via Dll1 and Dll4. This is especially important when considering the translatability of findings from insult-naïve, specific pathogen free mice to adult humans and other animals that have substantial pools of tissue-resident lymphocytes and have epigenetic memories of these past insults.

DLL/Notch signaling is, however, just one of many tools fibroblasts possess to restimulate infiltrating T cells. Fibroblasts in both SLO and non-lymphoid tissues express alarmins like IL-33 and Thymic Stromal Lymphopoietin (TSLP). ^67^^,^^68^^,^^71^ IL-33 produced by SLO fibroblasts is important for activation and effector differentiation of alloreactive CD4 T cells in murine GVHD models and effector CD8 T cells in acute and chronic viral infection. ^68^^,^^71^ Recent findings have pointed to the importance of IL-33 in balancing between CD8 stem memory T cell expansion and effector differentiation in chronic viral infections and cancer ( Figure 4D). ^69^^,^^395^ In non-lymphoid tissues, IL-33 expressing fibroblasts appear to be enriched at interfaces between microanatomic compartments. These interfaces include airways and vasculature in the lungs, portal triads and vasculature in the liver, vasculature in adipose tissue, hair follicles and subcutaneous fascia in murine skin and the dural sinus in the meninges. ^67^^,^^72^ Successful cancer immunotherapies create specialized perivascular niches that likely allow asymmetric division of CD8 stem memory T cells to preserve stemness and proliferative capacity while simultaneously generating differentiated cytotoxic CD8 T cells. ^396^ It appears that IL-33 released by perivascular fibroblasts in chronically inflamed tissues balances effector differentiation and stemness of infiltrating CD8 T cells to perpetuate autoimmune tissue destruction or control of viral reactivation and cancer. More broadly, specialized fibroblasts at tissue interfaces (e.g. mucosal/epidermal barriers, blood/parenchyma, tissue/cavity, etc.) may, upon activation, express an array of molecules that contribute to the restimulation or continued activation of infiltrating or activated tissue resident T cells.

If specialized fibroblasts promote local reactivation and differentiation of T cells, can they do the same for B cells? Like what was described for T cells above, Dll1 provided by murine FRC promotes MZ B cell survival and plasma cell differentiation ( Figure 4E). ^291^ MZ B cells express both classical (MHC) and non-classical (CD1 family) antigen presentation machinery. ^291^^,^^397^^–^^399^ MZ-like B cells expressing CD1 family transcripts are found in RA synovial tissue. ^400^ Thus, FRC- or FDC-like fibroblasts in the RA synovium may provide DLL1/Notch2 signals that promote MZ-like B cell survival and plasma cell differentiation in this inflamed tissue. It’s unclear, however, if human FRC or FDC provide similar Dll1/Notch2 signals and, if so, whether this system promotes B cell survival. This may extend to other inflammatory diseases where large B cell infiltrates are common, such as DLE and SS, although this awaits experimental validation.

Follicular dendritic cells (FDC) are specialized fibroblasts found in B cell follicles in SLO. Immune complexes deposit onto FDC, which depends upon complement receptors 1 and 2 (CR1 and CR2) and, in secondary follicles, FcγRIIB expressed by FDC ( Figure 4F). ^215^^,^^401^^–^^409^ Recycling of immune complexes by FDC controls how long B cell responses last in the absence of fresh insults, and how frequently antigen is available to drive affinity maturation of B cells. ^215^^,^^405^^,^^410^ This function of FDC is important for germinal center reactions and the generation of functional, high-affinity antibodies. ^213^^,^^215^^,^^403^^,^^406^^,^^408^^,^^409^^,^^411^ Immune complex recycling by FDC also means that B cell activation and germinal centers can be maintained long after the initiating insult is cleared. ^401^^,^^402^^,^^404^^–^^406^^,^^410^ Do FDC-like fibroblasts perform similar B cell activating functions in tissues affected by IMID?

FDC-like networks are associated with activation-induced cytidine deaminase (AID) expression by accumulating B cells during inflammation in non-lymphoid tissues ( Figure 4F). ^21^^,^^159^^,^^162^ This local AID expression appears to be functional. The presence of secondary follicles in RA synovia, the lungs of RA patients with pulmonary complications and salivary glands of SS patients is associated with higher serum levels of anti-rheumatoid factor. DNA sequencing of B cells from germinal center positive joints suggests local clonal expansion and affinity maturation of autoantibodies. ^21^^,^^200^^,^^361^^,^^412^ Similarly, transplanting RA synovium samples either with or without CD21+ FDC into SCID mice demonstrated that AID expression and mature FDC were associated with local class switching and secretion of human anti-citrullinated protein IgG. ^210^ Murine models of infection and autoimmunity also indicate that B cells can undergo local antigen-specific proliferation and affinity maturation only in the presence of mature FDC ( Figure 4F). ^107^^,^^153^^,^^154^^,^^159^^,^^198^^,^^204^ Synovial levels of an FDC transcriptional signature and individual immune complex trapping genes were positively correlated with both DAS28-ESR and DAS28-CRP supporting the idea that local autoreactive B cell responses are associated with more aggressive or severe disease. ^303^

Local B cell activation by synovial fibroblasts recapitulates *in vitro.* Co-culture of naïve IgD+ B cells with synovial fibroblasts from RA patients induces AID expression, class switching to and secretion of IgG without any exogenous stimulus. AID expression as well as class switching to and secretion of IgA and IgG is enhanced by TLR3 stimulation during the co-culture. ^301^ TLR3-dependent induction of AID and class switching requires BAFF signalling through its receptors. ^301^ This appeared to occur independent of antigenic stimulation because combined TNFα and LTα1β2 signaling was necessary to increase transcripts of the immune complex trapping genes Complement receptors 1 and 2 (CR1/CD35 and CR2/CD21) and Fragment crystallizable gamma receptor IIb (FCGR2B) by RA synovial fibroblasts. ^303^ The requirement for combined TNFR and LTβR signaling in the full maturation of FDC is supported by *in vivo* pharmacological blockade and genetic disruption data in mice, rats and monkeys. ^145^^,^^192^^,^^194^^,^^197^^,^^202^^,^^413^^,^^414^ However, there may be other pathways to full FDC maturation as they form in inducible bronchus-associated lymphoid tissue in mice lacking TNFα, LTα and LTα1β2. ^198^

These observations are particularly relevant to developing treatments for patients with autoantibody or B cell-driven autoimmune diseases. First, these local B and plasma cell responses in IMID likely give rise to tissue resident B and plasma cells. ^208^^,^^415^^–^^418^ Rituximab appears to require B cells to recirculate for clearance via antibody-dependent phagocytosis or complement-dependent cytotoxicity, and thus fails to deplete tissue resident B cells. ^161^^,^^207^^,^^419^ Other therapeutic approaches including targeting survival factors that affect B cells and/or plasma cells in the bone marrow, SLO and TLS, depleting antibodies that work by antibody-dependent cellular cytotoxicity or chimeric antigen receptor T cell (CAR-T) or combination therapy approaches will likely be necessary for larger clinical benefit in these patients. ^420^^–^^423^ Second, because FDC trap and retain antigen for at least 56 days either clearance of immune complexes from FDC or depletion of FDC is necessary to prevent perpetuation or re-emergence of autoreactive B and plasma cell responses. ^215^

## Conclusions

Over the past 20 years, increasing interest in the roles that fibroblasts play in both FD and IMID has revolutionized our understanding of these cells. This growing body of work suggests that over the course of FD and IMID, fibroblasts in the affected tissues undergo progressively divergent differentiation which is associated with the distinct clinical outcomes of these diseases. It seems likely that acute injury signals including alarmins, PRR activation and the serum response initiate fibroblast activation early in both FD and IMID. While this has been demonstrated in several animal models of FD and IMID, conclusive proof from patients will require the development of diagnostics that can detect and differentiate these diseases before they manifest clinically. As the types of acute injury signals vary widely, future research into whether these differ between FD and IMID will help us better understand whether differences in fibroblast functions are initiated in the earliest stages of disease.

Currently, it seems that fibroblast activation by alarmins in FD and IMID translates to their recruitment of and growth factor support for monocytes and granulocytes in the early stages of disease. Several therapies targeting the alarmins that activate fibroblasts, among other cells, have entered the clinic or are under development and have met with varied success. IL-1 antagonists have primarily shown efficacy in IMID which have associated familial genetic mutations in pathways involving IL-1. Efficacy in IMID has been mixed, with promising results seen in idiopathic juvenile arthritis and hidradenitis suppurativa, both of which often present with substantial neutrophil infiltrates (reviewed in Ref. 424). The mixed success of IL-1 antagonists may, in part, reflect the redundant stimuli (IL-1, TNFα, serum, etc.) that drive production of neutrophil and monocyte chemokines, growth factors and activation molecules by fibroblasts. Combined blockade of these pathways may be necessary to drive clinical responses in a broader population of patients as was recently proposed. ^37^

After their initial activation by acute injury signals, divergence of fibroblast differentiation states between FD and IMID appears to be a sequential process due, in part, to an evolving dialogue with infiltrating leukocytes. This is supported by the distinct sizes and compositions of leukocyte infiltrates observed when FD and IMID patients present clinically. In IMID where there is a break in tolerance, autoreactive T cells infiltrating the affected tissues retain mature DCs presenting cognate antigen around blood vessels and sites of damage. Clustering of macrophages and DCs with T cells and other tissue resident lymphocytes may trigger subsequent fibroblast differentiation through combinations of TNFR1/2, LTβR and IL-22 signaling along with cytokines produced by type 1, type 2 or type 3 polarized immune cells. Ultimately, this can lead to TLS formation with local priming of autoreactive T cells and class switching, affinity maturation and autoantibody production by B and plasma cells.

The many therapies antagonizing TNFR1/2, IL-13 and IL-17 that have been approved for IMID give physician scientists opportunities to investigate whether any of these signals are required for the maintenance of lymphocyte aggregates or TLSs. As each of these has animal model and *in vitro* data supporting their direct effects on fibroblast differentiation and lymphocyte aggregate formation some of their efficacy may come from reversing this. Recently, RA pathotypes reflective of distinct fibroblast differentiation have been associated with the course of disease progression and clinical responses to different DMARDs. ^161^^,^^199^^,^^303^ Enriched efficacy of tocilizumab in RA patients with the diffuse myeloid pathotype fits with the hypothesis that trans-IL-6 signaling between these two cell types is pathogenic in many of these patients. ^161^ This observation also suggests that combined blockade of IL-6 receptor and an orthogonal pathway involved in lymphoid aggregate formation, like TNFR1/2 or LTβR, may increase the breadth of responding patients to those with the lympho-myeloid pathotype as was recently proposed. ^425^

The necessity of combination therapies is further reinforced by the coexistence of multiple fibroblast functional states in the same tissue. This has been best described in RA where fibroblasts specialized for supporting local autoimmune reactivation in lymphoid aggregates and erosive fibroblasts that degrade cartilage occur in the same tissue. Such functionally different fibroblasts arising in the same tissue suggests the existence of distinct microanatomic signaling niches within the tissue. Although targeting different therapeutics to microanatomic niches in the same tissue is beyond our current engineering capabilities, rational target combinations in multispecific large molecules or selectively engineered small molecule polypharmacology may serve as a bridge to the future. Eventually, combining multiple targeted lipid nanoparticle, RNA or small molecule therapies into a single formulation may achieve something resembling microanatomically directed therapies.

Parallel to the observation of these lymphoid niches in IMID is the finding of niches comprised of ECM fibroblasts, Fab5 macrophages and neutrophils in FD. In FD, acute injury activates fibroblasts to produce chemokines that attract neutrophils and monocytes. However, the absence of infiltrating T cells may lead to DC depletion, which allows PDPN+ fibroblasts to contract and stiffen the local ECM. This begins activating latent factors in the ECM, such as TGFβ. Simultaneously, these activated fibroblasts produce GM-CSF to keep infiltrating neutrophils and monocytes alive. Together with neutrophils and γδ T cells producing IL-17A, these activated fibroblasts induce monocytes to differentiate into Fab5 macrophages. ECM fibroblasts and Fab5 macrophages form CDH-11 synapses where the macrophages produce additional latent TGFβ for integrin-mediated activation by fibroblasts. Given the mixed type 2 and type 3 inflammatory tone in some FD it is likely that ECM fibroblasts are also activated by IL-4 and IL-13 produced by ILC2, granulocytes, iNKT and MAIT cells. These type 2 cytokines polarize macrophages away from the Fab5 profile and towards a remodeling state aimed at re-epithelialization and -vascularization.

Like our observation of the need for combination therapies in IMID, the coexistence of mixed inflammatory skewing in FD indicates a similar requirement for combination therapies. Blocking the key type 3 cytokines in some FD appears to drive a compensatory spike in type 2 inflammation. Conversely, inhibiting fibrotic type 2 signaling seems to induce a rebound in type 3 inflammation. Simultaneously inhibiting both systems may be necessary to cover broad FD patient populations. Similarly, as the RA community has begun to define pathotypes associated with distinct clinical outcomes and responses to therapies, FD researchers and clinicians will benefit from more detailed characterization of disease heterogeneity in their patients. Additionally, as the inflammatory phase of FD appears to precede end-stage fibrosis the development of diagnostic methods to identify and classify these patients earlier in their disease progression will likely improve the frequency of successful therapeutic intervention.

Finally, the observation of local, durable immune responses in tissues affected by FD and IMID has consequences for depleting therapies. For example, the lympho-myeloid RA pathotype is the only one with large B cell infiltrates in the affected joints. Why, then, was this pathotype not enriched for rituximab responders? ^161^ Possibly because rituximab was unable to deplete synovial B cells due to a lack of recirculation or low levels of complement-mediated lysis in the joint. Similarly, the niche in FD appears to create a similar challenge for conventional depleting therapies. Three therapeutic strategies currently under investigation may address this challenge.

First, activated cells, whether lymphocytes or fibroblasts, appear to be more susceptible to pharmacological perturbations of the balance between pro- and anti-apoptotic proteins. ^295^^,^^426^ Better understanding how these differ between activated fibroblasts in FD and IMID may allow successful small molecule or RNA targeting of these systems. Second, depleting antibodies with Fc regions engineered for efficient antibody-dependent cellular cytotoxicity may circumvent the need for phagocytosis of opsonized cells by Kupffer cells or red pulp macrophages. Third, CAR-T or other engineered cell therapies can allow for targeted depletion of pathogenic cell types throughout the body as recently demonstrated in systemic lupus erythematosus and myasthenia gravis patients, and animal models of cardiac fibrosis. ^420^^,^^427^^,^^428^ Regardless of the therapeutic approach, we predict that accounting for and disrupting the fibroblasts that lay the foundation for pathogenic niches in FD and IMID will be critical for future therapeutics.

## Data Availability

No data are associated with this article.
