# Supplementary material for: Distinct fibroblast functions associated with fibrotic and immune-mediated inflammatory diseases and their implications for therapeutic development
Source: F1000Res. 2024 Jan 10;13:54. [Version 1] doi: 10.12688/f1000research.143472.1 (PMC11053351; doi:10.12688/f1000research.143472.1)
Supplement: Peer review history

# Reviewer report: Reviewer response for version 1

**DOI:** 10.5256/f1000research.157138.r251130

**Published:** 2024-04-26

**Authors:** Theresa T. Lu, Mir Howlader

**Affiliations:** 1 Autoimmunity and Inflammation Program and Pediatric Rheumatology, Hospital for Special Surgery, New York, New York, USA; 2 Biochemistry, Cell and Molecular Biology, Weill Cornell Medical College, New York, New York, USA

Reviewed by Mir Howlader; 

 This review is very comprehensive, covering the distinct fibroblast functions in immune mediated inflammatory diseases (IMID) versus fibrotic diseases (FD). It discusses how disease initiation involves many different alarmins that lead to leukocyte infiltration and fibroblast activation. The review further explains how fibroblasts control the activation, differentiation, and survival of infiltrating leukocytes. It presents in depth the types and quantity of the growth factors fibroblasts produce and the various signals that can lead to differentiation of leukocyte subsets and formation of osteoclasts. It also discusses the pathogenic function of fibroblasts in the production and organization of the extracellular matrix (ECM), leading to fibrosis. Through every topic they explore, they discuss the potential therapeutic targets, the ongoing therapy research and they also address current gaps in the field and provide multiple experiments to try to fill in these gaps.

 I have a few minor suggestions:

- Consider adding sub-subheadings to the current 4 subheadings. For example, for the second subheading: Rationing of growth factors, it might be worth further breaking it down to “Growth factors involved in T cell survival in FD”, “Growth factors involved in T cell survival in IMID”, Growth factors involved in B cell survival in FD”, Growth factors involved in B cell survival in IMID”. It is mostly written in these subdivisions already but just adding a heading will help readers orient themselves better and then also refer back to specific topics more easily.
- Subheading 4 is in a different format, font, and color from the other subheadings.
- Figure 1 is great but just a little difficult to follow in terms of the cytokines, what they are doing and who is producing them. It might be helpful to add arrows to make more clear which signals are activating the fibroblasts and which signals the fibroblasts are producing.

Reviewed by Theresa Lu; in agreement with Mir Howlader's review of this outstanding manuscript. This paper will be a valuable resource across multiple fields.

Is the review written in accessible language?

Yes

Are all factual statements correct and adequately supported by citations?

Yes

Are the conclusions drawn appropriate in the context of the current research literature?

Yes

Is the topic of the review discussed comprehensively in the context of the current literature?

Yes

Reviewer Expertise:

Immunology, Autoimmune disease - lupus, Stromal cells in skin and lymph nodes, Lymphatics

We confirm that we have read this submission and believe that we have an appropriate level of expertise to confirm that it is of an acceptable scientific standard.

# Reviewer report: Reviewer response for version 1

**DOI:** 10.5256/f1000research.157138.r259951

**Published:** 2024-04-09

**Authors:** Hao Li

**Affiliations:** 1 Harvard Medical School, Boston, MA, USA

In this manuscript, authors aimed to provide a comprehensive summary on fibroblast functions. However, the organization of the paper is a little bit hard to follow. Also, if authors can deliver the concepts more clearly and concisely, it will make the paper more readable.  

 Main concerns,

1. The introduction is interesting but not appropriate. Keeping language formal in scientific writing helps maintain professionalism.
2. Fibroblasts are a type of cells which play important roles shaping our tissues. It is obvious that fibroblasts in lymphoid organs and non-lymphoid organs have distinct functions under naïve status.  Recent advances of application of single-cell RNA-seq provides solid evidence on fibroblast heterogeneity.  A brief introduction of fibroblast heterogeneity at the beginning will help readers better understand their distinct functions.
3. Describing the function changes of fibroblasts from naïve status to disease status will provide a better readable synthesis.
4. Sometimes, subtitles are not consistent or fully supported by the following paragraphs. For example, except producing chemokines, there are other mechanisms through which fibroblasts help positioning or organizing immune cells such as expressing adhesion molecules.
5. Fibroblasts are not only the responders to alarmins but also the main producers of almonds. For example, it is generally accepted that stromal cells are the major producers of IL-33.

Minor concerns,

1. It is not proper to keep questions for readers without giving answers or related hypothesis. For example, *“*Is there evidence that fibroblasts are impacted by this secreted form?”
2. Proper punctuation needs to be checked before submission.
3. * A*bbreviations must be spelled out completely on initial appearance in the manuscript.
4. Every statement needs to be supported by properly cited studies.  

Is the review written in accessible language?

Yes

Are all factual statements correct and adequately supported by citations?

Partly

Are the conclusions drawn appropriate in the context of the current research literature?

Yes

Is the topic of the review discussed comprehensively in the context of the current literature?

Yes

Reviewer Expertise:

Immunology and rheumatology

I confirm that I have read this submission and believe that I have an appropriate level of expertise to confirm that it is of an acceptable scientific standard, however I have significant reservations, as outlined above.

# Reviewer report: Reviewer response for version 1

**DOI:** 10.5256/f1000research.157138.r251132

**Published:** 2024-03-29

**Authors:** Kevin Wei

**Affiliations:** 1 University of California Irvine, Orange, California, USA; 2 Harvard Medical School, Boston, USA; 3 Oregon Health & Science University, Portland, OR, USA

Fibroblasts and important cells in chronic inflammatory diseases and fibrotic diseases.  Overall, this review is well-written and covers recent advances in our understanding of fibroblast pathology in the setting of chronic inflammatory diseases. 

 Major comments:

1. Fibroblast nomenclature.  Throughout the review, fibroblasts are mostly described as a total population.  In some discussions, specific fibroblast subtypes are discussed as “adventitial”, or “activated”.  Given recent advances in our understanding of fibroblast heterogeneity by scRNAseq, the authors could consider describing these specific subtypes in terms of markers that have been used to track these populations, especially in human disease settings.  For example, are adventitial fibroblasts PI16+/CD34+ universal fibroblast?  Are “activated” fibroblasts CXCL12+ or CCL19+ fibroblast as described in chronic inflammatory diseases?  An effort to link descriptive terms to molecular markers would help the readers better understand the existing literature on fibroblast heterogeneity.   This comment applies to Figure 2 as well.  Would consider adding key markers that distinguish these fibroblast subtypes. 
2. The introductory paragraph is colorful, although not completely appropriate for this scientific review.  I recommend removing the first paragraph.   
3. The statement “IMID generally features  lymphocyte rich infiltrates and aggregates” requires citation.  This statement is also not completely accurate as many auto inflammatory diseases are characterized by myeloid and neutrophil predominance in the affected tissues.  The authors should consider discussing IMID into separate conditions by autoimmunity (i.e. RA and SLE) vs auto inflammatory diseases (Stills’s, JIA, etc).   These conditions have distinct involvement of the adaptive and innate immune system. 
4. During the discussion of alarmins and the detection of cytokines from patient samples (page 3, and page 5), the authors should clarify and separate studies which measure these cytokines in the periphery vs those studies which measure the expression of cytokines directly in affected organ tissues.    
5. The discussion of combination therapy (page 5) in IBD is of interest.  However, it would be important to mention previous clinical trials involving biologics targeting multiple cytokines and discuss relevant clinical evidence for combination therapy in IMIDs.     
6. It would be helpful to the readers to understand key differences and similarities in function between human fibroblasts in the context of IMID vs fibroblasts in animal models of inflammatory diseases.  This is critical to this review as much of the discussion is centered on targeting fibroblasts in IMID, and pointing out differences in animal models and human diseases is highly relevant.  For example, fibroblast contribution to CXCL13 in mouse vs human. 
7. In Figure 2, the authors may consider illustrating distinct fates of fibroblast activation, modulated by inflammatory cytokines, growth factors, and niche signals.  It is not entirely clear by the current scheme how many distinct fibroblast outcomes. 
8. The term “ECM fibroblast”  can be used to describe both fibro genic properties – laying down ECM, and ECM modulating properties (i.e. breaking down matrix).  Do the authors intend to use the term “ECM fibroblast” broadly to describe both properties, or would the authors consider describing these distinct properties as “fibrogenic” vs “ECM degradation”?

Minor points:

1. Page 5: “”Does this mean that fibroblasts express nuclear IL-33 but cannot secrete it?”  Recommend avoiding rhetorical questions if they are not followed by a discussion.
2. Page 5: redundancy instead of “redunance”

Is the review written in accessible language?

Yes

Are all factual statements correct and adequately supported by citations?

Yes

Are the conclusions drawn appropriate in the context of the current research literature?

Yes

Is the topic of the review discussed comprehensively in the context of the current literature?

Yes

Reviewer Expertise:

fibroblast biology, genomics, organoids

I confirm that I have read this submission and believe that I have an appropriate level of expertise to confirm that it is of an acceptable scientific standard, however I have significant reservations, as outlined above.
